# Maternal cafeteria diet exposure primes depression-like behavior in the offspring evoking lower brain volume related to changes in synaptic terminals and gliosis

**DOI:** 10.1038/s41398-020-01157-x

**Published:** 2021-01-14

**Authors:** Luis A. Trujillo-Villarreal, Viktor J. Romero-Díaz, Iván Alberto Marino-Martínez, Lizeth Fuentes-Mera, Marco Antonio Ponce-Camacho, Gabriel A. Devenyi, M. Mallar Chakravarty, Alberto Camacho-Morales, Eduardo E. Garza-Villarreal

**Affiliations:** 1 Department of Biochemistry, College of Medicine, Universidad Autónoma de Nuevo León, San Nicolás de los Garza, NL México; 2 Neurometabolism Unit, Center for Research and Development in Health Sciences, Universidad Autónoma de Nuevo León, San Nicolás de los Garza, NL México; 3 Gene therapy Unit, Center for Research and Development in Health Sciences, Universidad Autónoma de Nuevo León, San Nicolás de los Garza, NL México; 4 Servicio de Anatomía Patológica y Citopatología. Hospital Universitario Dr José Eleuterio González, Universidad Autónoma de Nuevo León, San Nicolás de los Garza, NL México; 5 Cerebral Imaging Centre, Douglas Mental Health University Institute, Montreal, QC Canada; 6 Department of Psychiatry, McGill University, Montreal, QC Canada; 7 Department of Biological and Biomedical Engineering, McGill University, Montreal, Canada; 8 Instituto de Neurobiología, Universidad Nacional Autónoma de México campus Juriquilla, Queretaro, Mexico

**Keywords:** Molecular neuroscience, Depression

## Abstract

Maternal nutritional programming by caloric exposure during pregnancy and lactation results in long-term behavioral modification in the offspring. Here, we characterized the effect of maternal caloric exposure on synaptic and brain morphological organization and its effects on depression-like behavior susceptibility in rats’ offspring. Female Wistar rats were exposed to chow or cafeteria (CAF) diet for 9 weeks (pre-pregnancy, pregnancy, and lactation) and then switched to chow diet after weaning. By postnatal day 60, the male Wistar rat offspring were tested for depressive-like behavior using operational conditioning, novelty suppressed feeding, sucrose preference, and open-field test. Brain macro and microstructural morphology were analyzed using magnetic resonance imaging deformation-based morphometry (DBM) and western blot, immunohistochemistry for NMDA and AMPA receptor, synaptophysin and myelin, respectively. We found that the offspring of mothers exposed to CAF diet displayed deficient motivation showing decrease in the operant conditioning, sucrose preference, and suppressed feeding test. Macrostructural DBM analysis showed reduction in the frontomesocorticolimbic circuit volume including the nucleus accumbens (NAc), hippocampus, and prefrontal cortex. Microstructural analysis revealed reduced synaptic terminals in hippocampus and NAc, whereas increased glial fibrillary acidic protein in hippocampus and lateral hypothalamus, as well as a decrease in the hippocampal cell number and myelin reduction in the dentate gyrus and hilus, respectively. Also, offspring exhibited increase of the GluR1 and GLUR2 subunits of AMPA receptor, whereas a decrease in the mGluR2 expression in hippocampus. Our findings reveal that maternal programming might prime depression-like behavior in the offspring by modulating macro and micro brain organization of the frontomesocorticolimbic circuit.

## Introduction

Depression is one of the leading causes of disability worldwide affecting >300 million people of all ages^1,2^. Depressive subjects show anhedonia or low motivation for natural or social stimuli, which become resistant to brain therapy and classical pharmacology approaches^3^.

Major depressive disorder (MDD) is characterized by an age-dependent brain dysfunction and structural alterations in selective regions of the reward circuit^4–9^. The reward circuit integrates dopaminergic neurons located in the ventral tegmental area (VTA) that innervate the nucleus accumbens (NAc), the prefrontal cortex (PFC), central, and basolateral amygdala (BLA) and the hippocampus and dorsal striatum^10^. Glutamate neurons also originating in the VTA and substantia nigra (SN) of the midbrain innervate limbic sites, including the NAc and dorsal striatum^11–13^. Major volume brain changes in adult and adolescence in MDD subjects have been documented, including hippocampus atrophy^7,14^ thinner cortical gray matter in the orbitofrontal and medial cortex (OFC), anterior and posterior cingulate, insula, and temporal lobes^9,15–17^. Of note, genome-wide association studies (GWAS) of MDD and schizophrenia cohorts have identified genetic variants linked to brain volume alterations during development^18^, supporting the notion that brain macrostructural changes might potentially lead to MDD.

Aberrant brain morphological organization has also been observed in murine models of depression-like behavior. For instance, depression-like behavior models in rats show microstructural alterations linked to a decrease in hippocampal synaptophysin and NR2A subunit^19^, and dendritic atrophy in the CA1 and CA3 regions of the hippocampus^6,10,20–23^. Also, GWAS analysis of MDD and schizophrenia cohorts identified single-nucleotide polymorphisms in genes that encode synaptic plasticity and myelin repair proteins^18^. In addition to macrostructural and microstructural alterations of the reward system identified in MDD subjects or murine models, neurobiological causes underlying aberrant brain plasticity are unknown.

Epidemiological data and basic research studies in humans and animal models, respectively, have identified that maternal obesity^24,25^ and/or hypercaloric diet exposure during embryonic development^26–29^, a physiological process known as maternal programming, modulates establishment of functional and structural neuronal connectome of the reward system^27,28,30^, potentially leading to depression susceptibility in the offspring^26–29^. For example, maternal nutritional programming by exposure to a hypercaloric diet during pregnancy primed an altered glutamatergic neurotransmission in the reward system^28,30,31^, disruption in structural and functional integrity of the hippocampus^32,33^ and reduction in dendritic complexity in BLA of the offspring^33^. In humans, prolonged consumption of caloric diets during adolescence favors defective emotional behaviors^11,34^, which correlates with failure in hippocampal neurogenesis in murine models^32,35^. In this study, we hypothesized that maternal programming by hypercaloric diet exposure would produce macro- and microstructural alterations linked to brain volume changes and aberrant glutamatergic synaptic plasticity of the reward circuit in the offspring, leading to depression-like behavior early in life. For this, we studied behavior and global structural changes using magnetic resonance imaging (MRI), coupled with selective molecular and histological characterization of glutamatergic synaptic markers.

## Materials and methods

A full description of all experimental procedures is provided in the Supplemental Materials.

### Animals and housing

Programing and mating experiments were performed using males and virgin females from 10 to 12 weeks old Wistar rats, respectively. Animals were handled according to the NOM-062-ZOO-1999 guide for the care and use of laboratory animals, with approval of the Universidad Autónoma de Nuevo León Animal Care Committee (BI0002) (Supplemental information).

### Maternal nutritional programming model in offspring by cafeteria (CAF) diet exposure

Female Wistar rats were fed with chow (Control) or CAF diets for 9 weeks (pre-pregnancy, pregnancy, and lactation) as reported^24^. CTRL-CTRL and CAF-CTRL offspring were fed with control diet and CAF-CAF offspring were fed with CAF diet after weaning at postnatal day 21 (Fig. S1). Control chow and CAF diet formulas and caloric density are found in Table S1. At 2 months of age, we performed behavioral tests to characterize motivation. We registered body weight of all offspring at birth (~15 rats/litter) and at the age of 3 weeks, we killed female offspring. Body weight were quantified in offspring from 3rd to 7th week.

### Depression-like behavioral phenotyping

Individual animal behavior analysis was conducted on two cohorts, the first one for MRI, immunohistochemistry, and histology, and the second one for western blot analysis of synaptic markers (Fig. S2). For behavioral phenotyping we used the Skinner box for operational conditioning, as previously reported^28,36^, the preference to sucrose test, novelty suppressed feeding and open-field test as described in Supplemental information

### Ex-vivo fixation and MRI analysis by deformation-based morphometry (DBM)

Rats were anesthetized with 1 mL pentobarbital (PiSA Agropecuaria) i.p. overdose and transcardially perfused following standardized methods as described in Supplemental information. For MRI acquisition, the skulls were submerged and fixed inside plastic tubes filled with Fomblin (a chemically inert perfluoropolyether fluorocarbon; Solvay Solexis, Inc.) and imaging was performed in a 16 cm bore 7 T Bruker scanner, T1w sequence name: UANL_Camacho_FatRat, resolution 1.25 mm (Pharmascan 70/16), Bruker FLASH, slice thickness = 0.0853 mm, TR/TE = 30.76/8.64 ms, flip angle = 20 degrees, averages = 1, matrix = 376 × 376, spacing = 0.0853 mm, Pixel bandwidth = 74 Hz, FOV = 300 × 300 mm, no. of slices = 376. Morphological analysis was performed by converting DICOM to MINC format, and then preprocessed using an in-house pipeline based on MINC-Tools and the pydpiper pipeline (https://github.com/Mouse-Imaging-Centre/pydpiper) (see Supplemental information). All analyses were performed using pydpiper^37^, R statistics^38^, R studio^39^, and the RMINC^40^ and tidyverse^41^ packages.

### Histological analysis

Following MRI analysis, coronal sections from the brains were cut in a cryostat and stained with Hematoxilyn & Eosin, Kluver–Barrera stain, and synaptic markers were also evaluated through immunohistochemistry (Supplemental information).

### Membrane and cytoplasmic fractions isolation from the brain samples

Brains from the second cohort of subjects were dissected from the skull and the NAc, the PFC and the hippocampus were isolated using the Paxinos and Watson atlas (AP 1, 3, and 3.8 mm from bregma, respectively) as we reported previously^32^ (Supplemental information).

### Western blot analysis

Samples were subjected to sodium dodecyl sulfate polyacrylamide gel electrophoresis to identify changes in synaptic markers including NMDA, AMPA, synaptophysin, mGlur2, and Glur5 (see Supplemental information for details).

### Quantification and statistical analysis

Data are presented as mean ± SEM for all data. All statistical analyses including testing the normality of data distribution were performed using GraphPad Prism 7.01 and IBM SPSS statistics version 22 software and a corrected *p* value <0.05 was considered as significant. All results were tested for normality using Shapiro–Wilk test. For differences between three groups in the behavioral tests and protein concentration one-way Analysis of variance (ANOVA) followed by Tukey’s multiple comparison test was used and effect size was calculated in R language with the pwr package. For significant differences in high vs low responders during operant conditioning we used Chi-square per sample test. The data are shown as the mean ± SEM and significant differences *p* < 0.05. The statistical analysis on MBD was performed using the log-transformed Jacobian determinants as the dependent variable, “group” as the independent variable (between subjects) and as covariate we included “batch order” (rats were trained in batches). We compared the three groups using a GLM and analyses were corrected for multiple comparisons using the false-discovery rate (FDR) at 5%^42^. Furthermore, we extracted the *t* alues from significant peaks at NAc and hippocampus to create scatterplots and correlation with the immunohistochemistry data. Details about the statistical analysis are available in the open-access R script (see above).

## Results

**Fig. 1:**
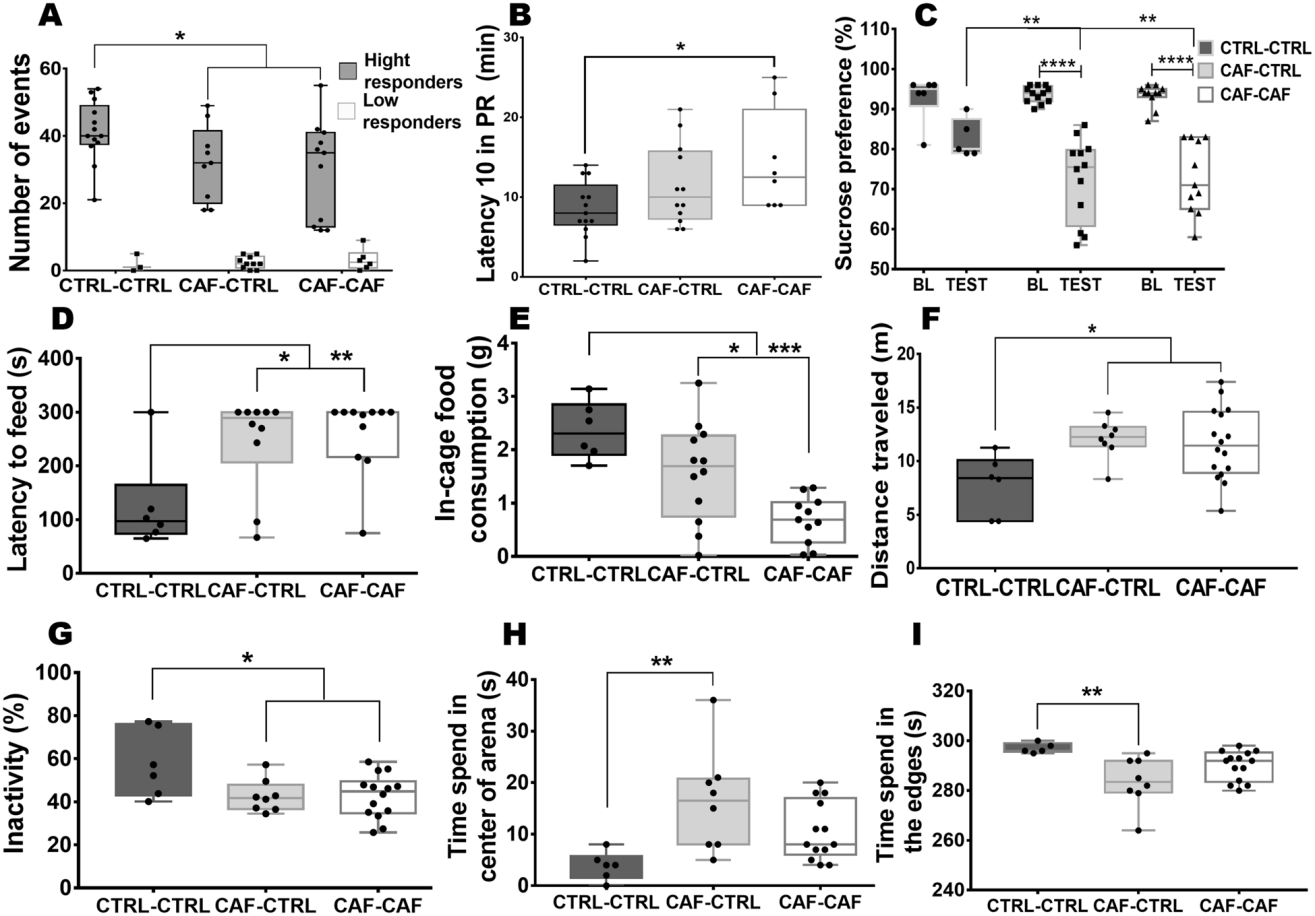
Behavioral testing of depression-like behavior in the offspring. **A** Offspring was nutritionally programmed as described, and subjects were trained using the operational conditioning protocol. The number of events (action of the lever) were obtained during the PR protocol (1 h × 5 days). Graph shows response to reinforcers of two subject groups, high responders (>10 lever presses) and low responders (<10 lever presses) per training session. Results are expressed as mean ± SEM, following by ANOVA two ways, Post hoc Tukey. *p < 0.05 vs the control. n = 16–19/group). **B** Latency to PR. Graph shows the time the subject invests to reach the threshold of 10 events during the PR schedule. Results are expressed as mean ± SEM, following by ANOVA two ways, Post hoc Tukey. *p < 0.05 vs the control. *n* = 16–19/group). **C** Basal sucrose intake was measure for 72 h under ad libitum food and water exposure. Sucrose preference was performed by quantifying water and 2% w/v sucrose intake for 20 min after food and water deprivation for 16 h. The percentage of preference for sucrose in the offspring was quantified according to % PS = [IS ÷ (SI + WI)] × 100. Results are expressed as mean ± SEM, following by ANOVA post hoc Tukey. ****p < 0.0001 vs the control. *n* = 6–12/group. **D** Latency to feed was determine comparing time required to reach the food of the center of the arena in the Control, CAF-CTRL and CAF-CAF groups. Results are expressed as mean ± SEM, following by ANOVA post hoc Tukey. *p < 0.05 **p ≤ 0.01 vs the control. *n* = 6–12/group. **E** Food intake in cage after fasting in the CAF-CTRL, CAF-CAF, and control groups. Results are expressed as mean ± SEM, following by ANOVA post hoc Tukey ***p < 0.05 vs the control. *n* = 6–12/group. **F**–**I** Comparison of total distance traveled, inactivity and time spend in on the edges and in the center of the open-field test. Results are expressed as mean ± SE following by ANOVA post hoc Tukey. *p < 0.05, **p < 0.005, ***p < 0.001, ****p < 0.0001 vs the control group, *n* = 6–16/group.

Individual behavioral phenotyping was performed in two cohorts of subjects to determine the effect of nutritional programming on motivation for rewards in offspring. Initially, we found that nutritional programming by CAF diet decreased offspring weight (Fig. S3; *F*_2,49_ = 20.88, *p* = 0.0005). only if the offspring continues the CAF diet after weaning (Fig. S3, *p* ≤ 0.0001). Motivation for natural rewards in rats displayed low and high responder subjects (Fig. 1A; F_2,46_ = 112.2, *p* = 0.0001), similarly as we reported recently^23^. In contrast to control subjects, fetal programming by CAF diet exposure decreases the motivation of high responder subjects (Fig. 1A; CTRL-CTRL vs. CAF-CTRL*p = 0.0190, effect size result was 0.96). In fact, fetal programming and CAF exposure after weaning reproduces low motivation for rewards in the offspring (Fig. 1A; CTRL-CTRL vs. CAF-CAF *p = 0.0170, effect size result was 0.94). In addition, offspring exposed to CAF experiences a delay in the lever presses performance (Fig. 1B; *F*_2,30_ = 3.652, *p* = 0.0381) compared with the CTRL-CTRL (Fig. 1B; *p = 0.0316, effect size result was 0.87), showed only significant in offspring exposed to CAF after weaning (Fig. 1A; CTRL-CTRL vs CAF-CAF *p = 0.0316). Analysis of the total high and low responder subjects show no significant difference 53% high responder and 47% low responder CAF-CTRL (*X*^2;^; *p* = 0.8185), and 65% high responder and 35% low responder CAF-CAF (*X*^2^; *p* = 0.2253), on the other hand, control group show more high responder (81%) than low responder (19%)(*X*^2;^; *p = 0.01242) (Table S2). Differences in motivation behavior in the offspring were also identified during the sucrose preference test (Fig. 1C; F_2,46_ = 4.784 *p* = 0.0130), showing a significant decrease in the percentage of sucrose intake in the CAF-CTRL and CAF-CAF groups compared with their baseline (Fig. 1C, ANOVA post hoc Tukey ****p ≤ 0.0001) and a significant decrease in the CAF-CTRL and CAF-CAF compared with the CTRL-CTRL in the test day (Fig. 1C, *p* = 0.0015 and *p* = 0.0074, respectively). Next, we examined hyponeophagia by NSFT (Fig. 1D; F_2,24_ = 5.833, *p* = 0.0086) programmed offspring displays longer time to reach the pellet in the center of the arena (Fig. 1D; CAF-CTRL and CAF-CAF vs. CTRL-CTRL, *p = 0.0234 and **p = 0.0088), and also show a decrease in individual food consumption following 18 h fasting (Fig. 1E; F_2,26_ = 11.55, *p* = 0.0003) CAF-CTRL and CAF-CAF vs. CTRL-CTRL (*p = 0169, ***p = 0.0002, respectively). Finally, offspring exposed to CAF increased their locomotor activity (Fig. 1F; *F*_2,27_ = 4.551, *p* = 0.0198) in contrast with the CTRL-CTRL group (Fig. 1F CTRL-CTRL vs CAF-CAF *p = 0.0280 and CTRL-CTRL vs CAF-CTRL *p = 0.0288), less inactive (Fig. 1G; *F*_2,27_ = 4.551, *p* = 0.0198; CAF-CAF *p = 0.0235, CAF-CTRL *p = 0.0431 vs CTRL-CTRL) and only the programed offspring fed with chow diet after weaning, exhibited longer time in the center of the arena when compared with edges and control subjects (Fig. 1H, I; *F*_2,24_ = 5.815, *p* = 0.0087, *F*_2,24_ = 5.797, *p* = 0.0088, CAF-CTRL and CAF-CAF vs CTRL-CTRL **0.0063 and **p = 0.0069 respectively). These data propose that fetal programming decreases motivation for natural rewards and increases latency to feed in the offspring.

### Programmed offspring show brain macrostructural alterations

**Fig. 2:**
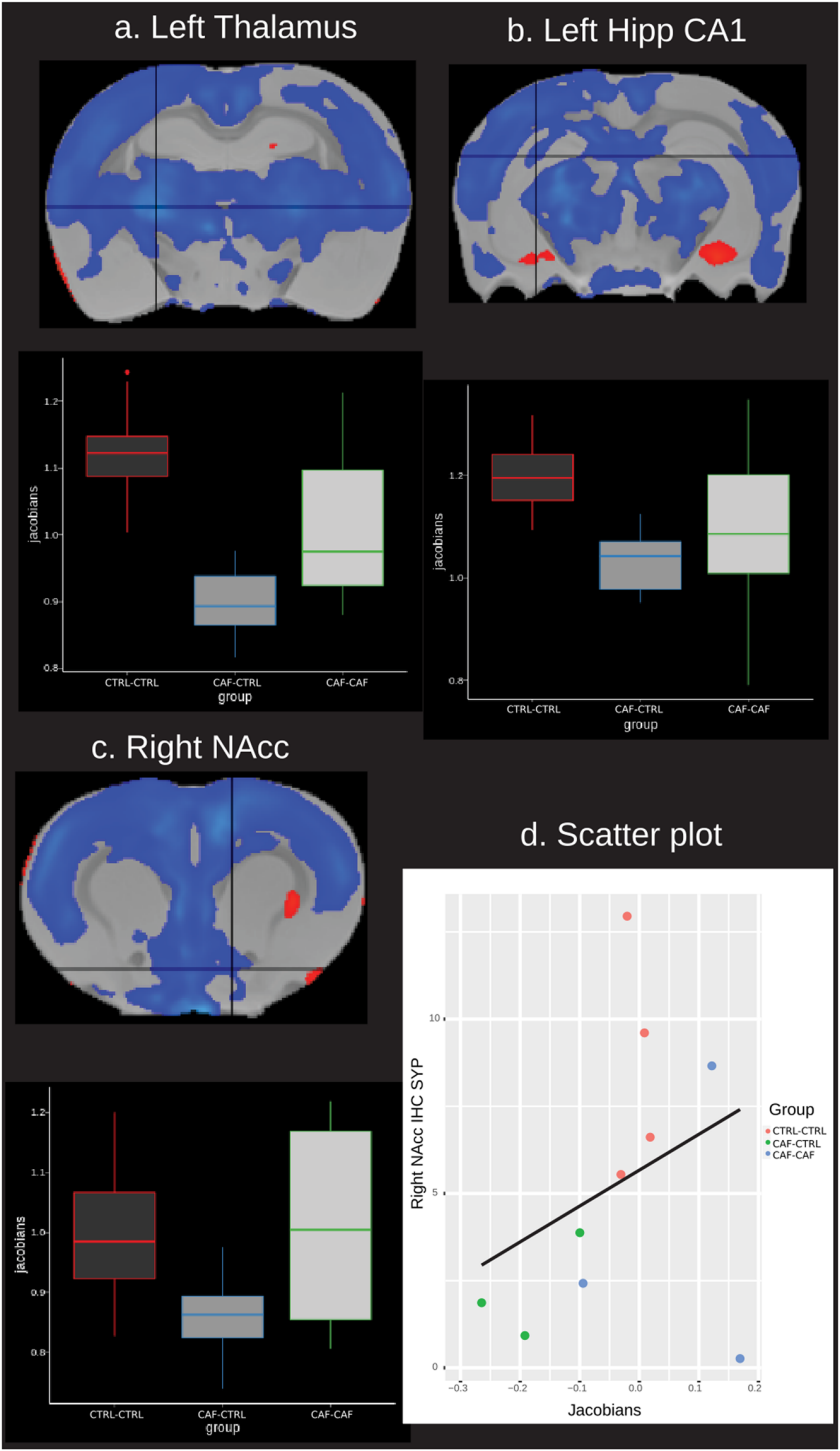
DBM of brain volume in the offspring. Boxplots show the relative volume (*y* axis = Jacobians) in each group (*x* axis) in several significant peaks. The peaks are shown in the crosshairs. **a** ROI = left thalamus, **b** left hippocampus CA1, **c** right NAc, **d** scatter plot between immunohistochemistry results in the right nucleus accumbens (*y* axis) and right nucleus accumbens peak volume (*x* axis). No significant correlation was found in **d**. Blue-light blue = lower volume; red-yellow = higher volume. Results are significant at FDR 5%.

We found significant differences in local volume across all groups. The pairwise analysis showed significant whole-brain local volume differences in the CAF-CTRL group compared with the CTRL-CTRL group (Fig. S4). The most consistent difference was lower local volume, however, there were localized clusters with higher volume (Table S3 and Table S4). The CAF-CAF group showed lower local volume compared with the CTRL-CTRL group in more localized clusters but only at FDR 10% and not 5%. There were no significant differences between CAF-CAF and CAF- CTRL groups. By using Fischer-344 rat anatomical atlas^43^, we identified that the left thalamus, left hippocampal CA1 and the right NAc core displayed the lowest volumes in the CAF-CTRL group. Notably, the peak volume of right NAc was proportionally correlated to synaptophysin expression (Fig. 2) (see data from immunohistochemistry below).

### Brain microstructural defects in offspring linked to fetal programming

**Fig. 3:**
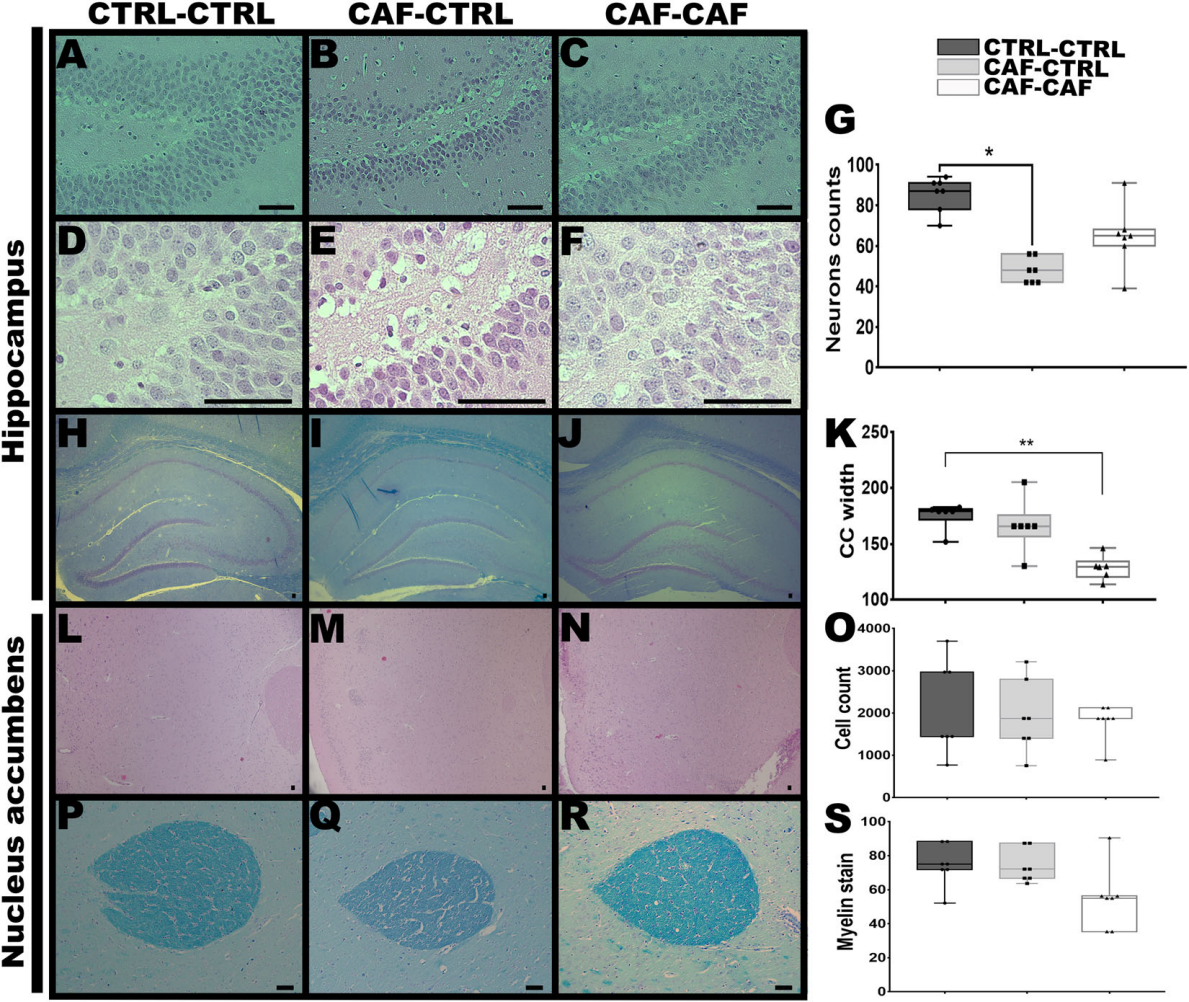
Histological analysis of hippocampus in the offspring. **A**–**G** Representative H&E stain of brain coronal slices comparing DG cellularity of the Control, CAF-CTRL, and CAF-CAF groups (400 and 1000 magnification). Results are expressed as mean ± SEM. following by ANOVA post hoc Tukey *p < 0.05 vs the control group, *n* = 4 per group). **H**–**K** Representative Luxol fast blue stain of brain coronal slices comparing corpus callosum of the CTRL-CTRL, CAF-CAF, and control groups (500 magnification). **L**–**O** Representative H&E stain of brain coronal slices of the NAc cellularity of the Control, CAF-CTRL, and CAF-CAF groups (400 magnification). **P**–**S** Representative histological image (coronal plane) of anterior commissure at NAc of the CTRL-CTRL, CAF-CAF, and control groups treated with luxol fast blue and cresyl-violet for myelin stain. Results are expressed as mean ± SEM. following by ANOVA post hoc Tukey. *P < 0.05, **P < 0.005, ***P < 0.001, ****P < 0.0001 vs the control group, (*n* = 4 per group). Scale bar = 50 μm.

We tested if maternal nutritional programming sets histological and synaptic protein expression defects in offspring. We specifically selected brain regions showing macrostructural alterations in the MRI analysis (Fig. S3, Table S3, and Table S4). First, hippocampal H-E staining identified a decrease in the total number of cell nucleus in the dentate gyrus (DG) of the offspring programmed by CAF diet (Fig. 3A–G; *F*_2,9_ = 5.978, *p* = 0.0223), showing only significant difference in CAF-CTRL compared to the CTRL-CTRL Fig. 3G *p = 0.0184), it also indicated pyknotic cells, chromatin condensation and cellular disorganization (Fig. 3A–G). Likewise, the Klüver–Barrera staining for myelin displayed that the offspring programmed by CAF diet and exposed to CAF diet after weaning showed a decrease in the area of corpus callosum at level of CA1 of hippocampus (Fig. 3H–K; *F*_2,9_ = 5.993, *p* = 0.0221, *p = 0.0267). Histological and myelin characterization of NAc does not show significant changes following maternal programming by CAF diet (Fig. 3L–S).

**Fig. 4:**
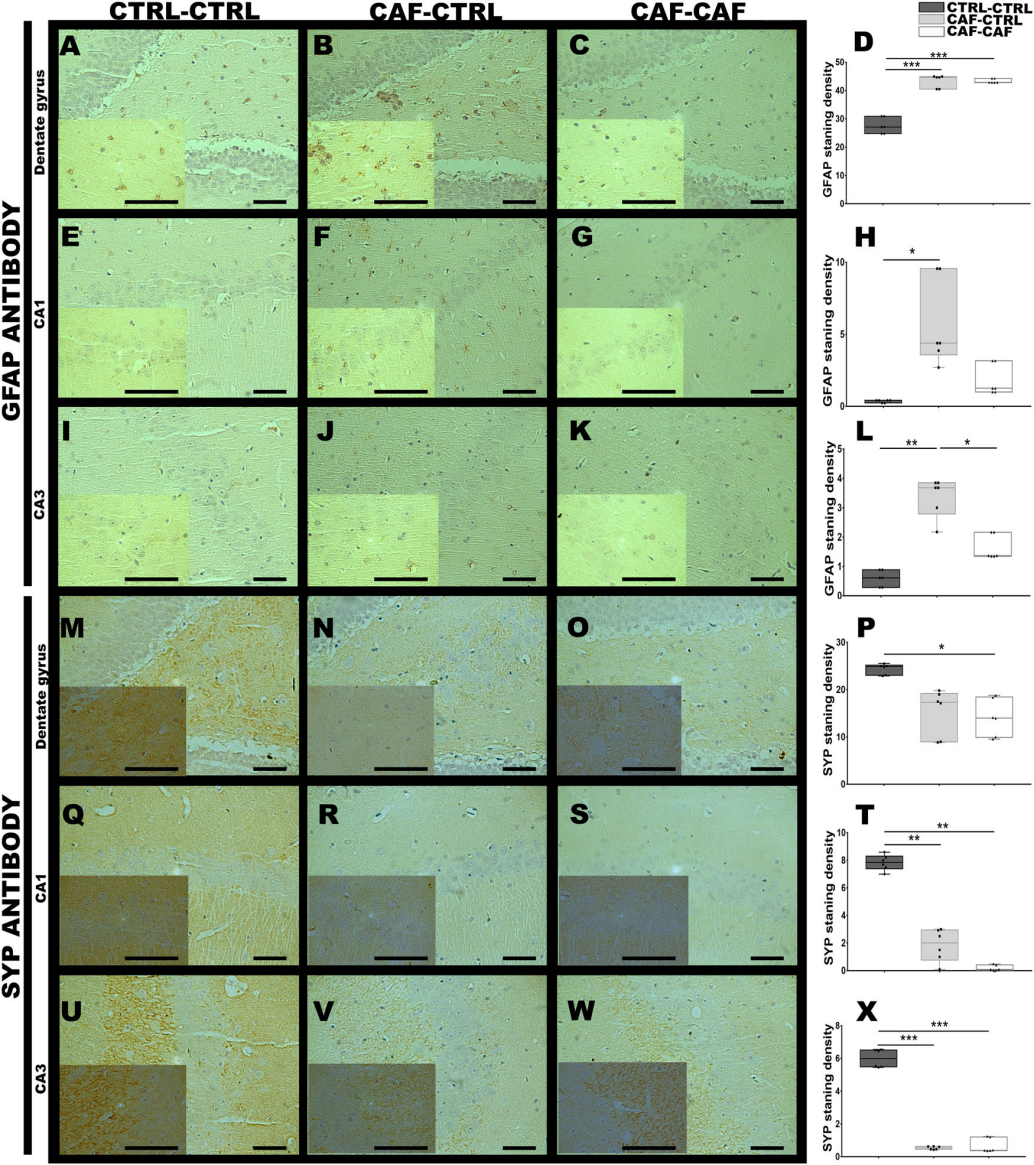
Maternal programming activates gliosis and decreases synaptophysin expression in depression-like behavior subjects. Coordinates for hippocampus was obtained from brain previously scanned by MRI. Brain slices were obtained as described in Methods. Immunostaining of sections using anti-GFAP antibody from dentate gyrus **A**–**D**, CA1 **E**–**H**, and CA3 **I**–**L** of hippocampal regions were tested for GFAP immunostaining. Immunostaining for synaptophysin was performed in hippocampus using an anti-SYP antibody of the Control, CAF-CTRL and CAF-CAF. **M**–**P** Dentate gyrus, **Q**–**T** CA1, **U**–**X** CA3 region. Results are expressed as mean ± SEM. following by ANOVA post hoc Tukey. *P < 0.05, **P < 0.005, ***P < 0.001, ****P < 0.0001 vs the control group (*n* = 4 per group). Scale bar = 50 μm.

Defects in synaptic markers in the offspring of mothers exposed to CAF diet were also determined using the subjects identified in the MRI analysis. Immunohistochemical imaging of brain regions showing macrostructural alterations revealed significant increase in the glial fibrillary acidic protein (GFAP) in the DG (Fig. 4A–D; *F*_2,6_ = 44.82, *p* = 0.0002), CA1 (Fig. 4E–H; *H*(2) = 7.318, *p* = 0.0043, *p = 0.0206) and CA3 (Fig. 4I–L; *H*(2) = 7.2, *p* = 0.0036, *p = 0.0219) regions of the hippocampus in subjects exposed to fetal programming by CAF diet (CAF-CTRL group, ***p = 0.0004, *p = 0.0206, and **p = 0.0036, respectively) and in the offspring exposed to CAF after weaning (CAF-CAF group, ***p = 0.0004, *p* = 0.5326 and **p = 0.0448, respectively)). Notably, a significant decrease in the synaptic marker synaptophysin in the DG (Fig. 4M–P; *F*_2,6_ = 5.606, *p* = 0.0424, CTRL-CTRL vs CAF-CAF *p* = 0.0497), and a substantial decrease in the CA1 (Fig. 4Q–T; *F*_2,4_ = 35.81, *p* = 0.0028, CTRL-CTRL vs CAF-CAF and CAF-CTRL *p* = 0.0027, *p* = 0.0074, respectively) and CA3 (Fig. 4U–X; *F*_2,4_ = 44.98, *p* = 0.0018, CTRL-CTRL vs CAF-CAF, and CAF-CTRL *p* = 0.0021, *p* = 0.0032, respectively) regions of hippocampus was identified in offspring of mothers exposed to CAF diet and in subjects exposed to CAF after weaning compared with the control. Also, there were no significant changes in the immunohistochemical stain of synaptophysin and no changes in the expression of GFAP marker in NAc when compared with the control group (Fig. S5A–H). Finally, the lateral hypothalamus showed a profound decrease in synaptophysin and GFAP immune signal in subjects exposed to fetal programming (Fig. S5I-P; *F*_2,7_ = 57.35, *p* = 0.0001, CTRL-CTRL vs CAF-CAF and CAF-CTRL, ****p = 0.0001 and ***p = 0.0001, respectively).

**Fig. 5:**
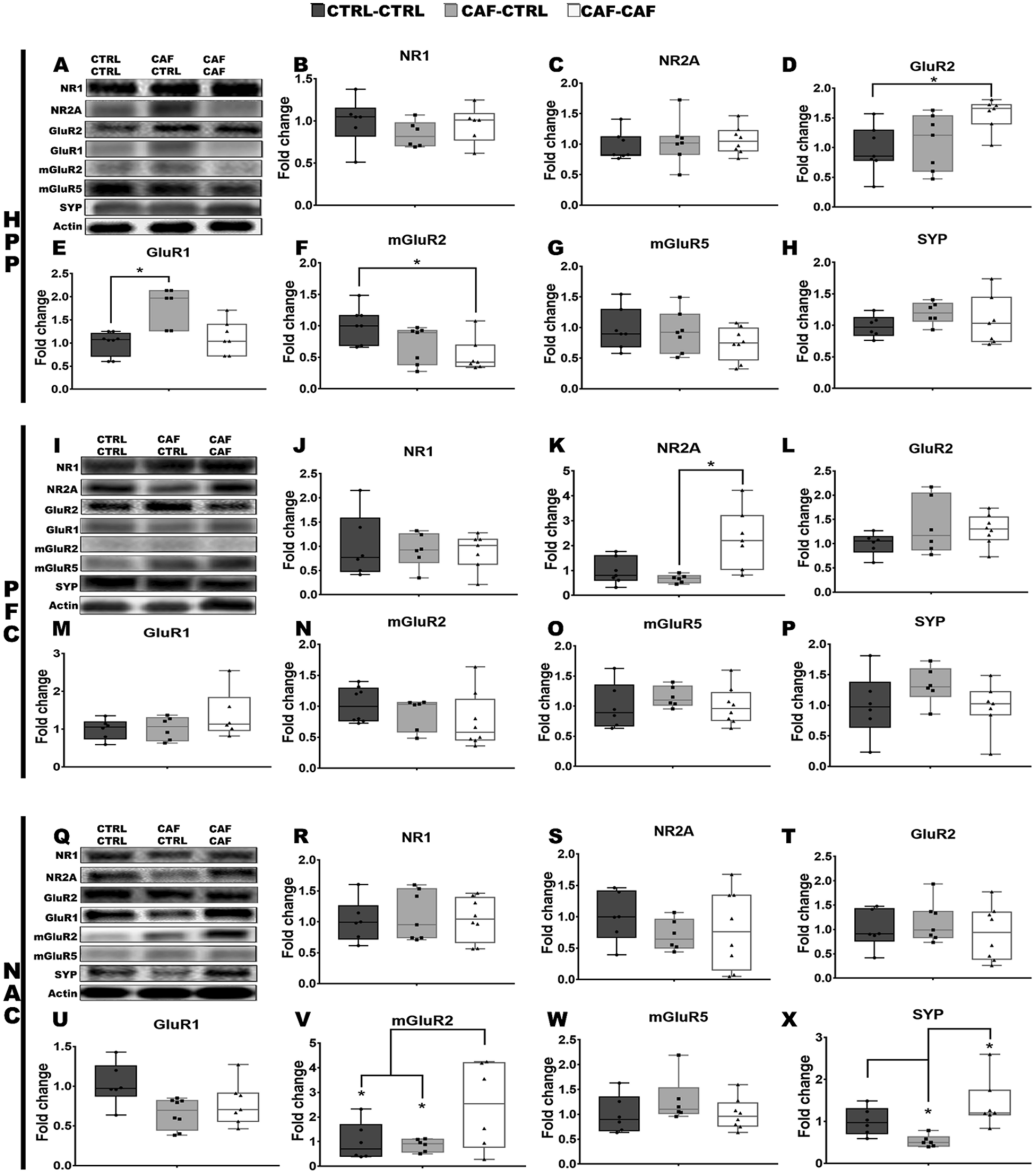
Characterization of the glutamatergic neurotransmission markers in depression-like behavior subjects. The second cohort of subjects was diagnosed as depression-like behavior and PFC, NAc, and hippocampus were isolated to perform western blot analysis for glutamatergic receptors markers including the NR1 and NR2A subunits (NMDA receptor), GluR2 and GluR1 subunits (AMPA receptor), mGluR2 and mGluR5 (metabotropic receptors) and synaptophysin (SYP). **A**–**H** hippocampus (HPP), **I**–**P** prefrontal cortex (PFC), and **Q**–**X** Nucleus accumbens (NAC). The graphs show normalized data of the mean ± SEM following by Tukey multiple comparation test. *p < 0.05 vs the control group. *n* = 5–8/group.

Synaptic defects of selective brain regions showing major structural changes evidenced by MRI analysis including hippocampus, PFC, and NAc in the offspring were detected by western blot analysis in the second batch of offspring subjects exposed to maternal programming. We focused our analysis on the glutamatergic neurotransmission markers including the NR1 and NR2A subunits (NMDA receptor), the GluR1 and GluR2 subunits (AMPA receptor), the mGluR2 and mGluR5 (metabotropic receptors) and synaptophysin for synaptic terminals (Fig. 5). Hippocampal analysis in the offspring of programmed mothers showed a significant increase in the GluR1 (Fig. 5E; *F*_2,9_ = 4.098, *p* = 0.0543, CAF-CTRL vs CTRL-CTRL *p = 0.0484) subunit protein expression, and an increase in the GluR2 (Fig. 5D; *F*_2,18_ = 4.626, *p* = 0.0239, CAF-CAF vs CTRL-CTRL *p = 0.0268) subunit as well as a decrease in the mGluR2 (Fig. 5D; F_2,16_ = 3.566, *p* = 0.0524, CAF-CAF vs CTRL-CTRL *p = 0.0428) expression in the offspring exposed to CAF after weaning. No changes were found in NR1, NR2A subunits, and mGluR5 and synaptophysin protein expression (Fig. 5B, C, G, H). PFC shows NR2A subunit upregulation in the offspring exposed to CAF after weaning (Fig. 5K; *F*_2,13_ = 4.477, *p* = 0.0332, CAF-CAF vs CAF-CTRL *p = 0.0310). Also, we did not find protein expression changes in NR1, GluR1, GluR2, mGluR2, mGluR5, or synaptophysin protein expression in PFC (Fig. 5J, L–P). Finally, NAc of programmed offspring subjects integrated a downregulation of synaptophysin in the offspring of mothers exposed to CAF diet (Fig. 5X; *F*_2,12_ = 7.408, *p* = 0.0080; CTRL-CTRL vs CAF-CTRL *p = 0.0407)), and an upregulation of mGluR2 and synaptophysin protein levels in the offspring exposed to CAF after weaning (Fig. 5V, X; *0.04901 and *p = 0.0144, respectively) (Fig. 5V, X). No changes were identified in the protein expression of NR1, NR2A, GluR1, GluR2, and mGluR5 (Fig. 5R, S, T, U, W). These results propose that caloric exposure during fetal development negatively regulates synaptic terminals expression in selective brain regions of the offspring.

## Discussion

Maternal overnutrition during pregnancy leads to several alterations in the offspring’s behavior such anxiety^44^, and addiction^28,30^, however, neurobiological causes of programmed offspring behavior induced by caloric exposure are not completely understood. Initially, we identified that maternal programming CAF-CAF diet exposure decreased body weight from week 4 to week 7 of age and the rats did not recover to control values (Fig. 1A), corresponding with our previous report^29^. We identified that maternal programming by CAF diet exposure led to depression-like behavior in the offspring, showing lower volume in many regions, among which the thalamus, hippocampus, NAc core, and hypothalamus, important regions in the frontostriatomesolimbic system, were correlated with their own alterations in SYP and GFAP expressions. Also, maternal programming led to a cell number and myelin reduction in the DG and hilus of hippocampus, respectively, and an increase in the GLUR1 receptor expression. This evidence proposes that maternal exposure to high-fat diet formula primes lower brain volume and changes in synaptic markers of glutamatergic neurotransmission which associates to depression-like phenotype in the offspring.

In accordance with a previous study^45^, our results show that fetal programming by CAF diet exposure during pregnancy contributes to hyperactivity in male offspring measured by distance traveled^46^. Following behavioral phenotyping of depression-like behavior for rats^47^, our results show for the first time that, despite no anxiolytic effect by fetal programming, the offspring experienced defects in motivation for natural rewards during the operant conditioning test, and a reduced preference for hedonic sucrose intake and latency to eat following fasting. In fact, defective motivation for natural rewards and anhedonia-like behavior have been reported in rats expose to chronic mild stress^20,48–53^, after chronic consumption of a high-fat food^11^ or even followed by exposure to sweet beverages^26,54^. In order to explain the effect of maternal programing of CAF diet exposure on depression-like behavior phenotype, we initially focused our analysis on aberrant brain microstructural alterations by MRI technology. Structural defects of selective brain regions are a hallmark of neuropsychiatric disorders including depression^55,56^, showing lower brain volume within the corticolimbic circuit diagnosed in the MDD subjects^57^. We found that maternal programming (CAF-CTRL group) led to dramatic brain volume loss in the left thalamus, left hippocampal CA1 and in the right NAc core. Also, the occipital cortex and the frontal association cortex displayed the major volume decrease of CAF diet programed offspring (CAF-CTRL group). Despite caloric exposure after weaning (CAF-CAF group), there were fewer brain volume changes when compared with the maternal programming group (CAF-CTRL), at least at this age. Depression-like behavior has been identified to show a time-dependent atrophy of CA1, CA3, and DG brain regions^50^, such as, reduced cortical thickness in prefrontal cortex and orbitofrontal, and smaller hippocampal volume and larger pallidal volume in children and adolescence diagnosed with MDD^9^. Conversely, increased cortical thickness in the bilateral posterior dorsolateral prefrontal cortex and right superior parietal cortex were found in MDD adult patients^15^. This evidence suggests a detrimental effect of caloric fetal programming on lower brain volume leading to depression-like behavior priming in the offspring.

To identify potential molecular or cellular causes of the lower brain volume measured with MRI in programmed subjects showing depression-like behavior, we initially focused our analysis in the lateral hypothalamus, based on its major brain volume loss. We found a direct decrease in GFAP levels and atrophy in the lateral hypothalamus of subjects exposed to CAF diet during pregnancy and lactation (CAF-CTRL group) and after weaning (CAF-CAF group). However, we did not find a significant correlation with MRI brain volume, potentially owing to low sample analyzed by immunohistochemistry. Our results agree with recent evidence showing a decrease in GFAP levels in hypothalamus following maternal high-fat diet exposure^58,59^, and also, deficient GFAP expression after immune activation by LPS inoculation^59–61^. We identified a correlation between the lower volume in the right NAc core and a decrease in synaptophysin immunosignal. However, in contrast to the lateral hypothalamus, we observed a prominent increase in the GFAP expression in the left hippocampal CA1 and the right NAc core. Selective increase in GFAP immunoreactivity was reported in layer I of the dorsolateral prefrontal cortex of brain post mortem biopsies of depressed subjects^62^. Conversely, studies in depressed post mortem patients^63^ and animal models of chronic stress reported a time-dependent reduction in glial cell number in the CA1 of hippocampus, potentially owing to a selective neuroadaptive response to stress^20^. Also, glial alterations in MDD might presumably be related to active release of S100B by astrocytes^64^ or microglia activation during programming^65^. Hippocampal shrinkage may be explained by a decrease in glial cells number and/or loss in the number of neurons due to a neurotoxic effect of glucocorticoids^66^, which are increased in depressed patients^4,67^, and have been found in murine models exposed to perinatal high-fat diet^68^. Similarly, we show that the hippocampus of offspring programmed by CAF diet displayed a decrease in cellular number in the DG coupled to pyknotic cells, chromatin condensation, cellular disorganization and a postweaning CAF diet intake decrease in the width of the body of the corpus callosum, which have also been implicated in MDD in humans^69–71^. Depression induced by chronic mild stress in murine models reported decrease cellular number and size in the CA1, thinner layers of cells in hippocampus^50^ and a decreased number of apical dendrites in CA1 and CA3^20^, which is also reported for mice or rats programming by a high-fat diet^33,35^. We conceive that an increase in the glial cells number found in our study potentially may compensate the synaptic loss and myelin decreases in the offspring showing anhedonia included in depression-like behavior.

Finally, we characterized microstructural changes of selected brain regions identified by MRI in subjects diagnosed with depression-like behavior by analyzing glutamatergic transmission markers which have been reported in both murine models and postmortem brains of depressive patients^12,28,48,72–74^. We found a substantial increase of the GluR1 and GLUR2 subunits of AMPA receptor in the hippocampus, which correlated with a low cell number at DG only in subjects programmed by CAF diet (CAF-CTRL group). Also, we found a decrease in the mGluR2 expression in hippocampus of subjects programmed by CAF diet and upregulation in NAc of subjects exposed to CAF diet after weaning (CAF-CAF group). Clinical observations in post mortem hippocampal samples from depressed subjects showed decreased expression of genes that encode AMPARs subunits^75^. A low expression of the subunit GluR1 and a high expression of the subunit GluR2, seem to be related to psychiatric disorders^76^. We speculate that a decrease of synaptic terminals evidenced by synaptophysin immunosignal following caloric exposure in offspring was compensated by upregulation of AMPA subunits expression. We have reported that glutamatergic receptor expression such as NMDA subunits become regulated by modifying in vivo glycolytic metabolism in hippocampus^77^, which also regulates neuronal death^78^. As reported, the offspring of mothers exposed to CAF diet integrates changes in the metabolic and hormonal plasma profile^29^, which correlates with metabolic defects found in a fetal programming model by high-fat diet exposure^79–81^, suggesting metabolic and hormonal molecular priming during embryonic development. In fact, we have recently reported that the offspring programmed by CAF diet show increased DNA methylation into the NAc^36^. This evidence supports “the fetal origins hypothesis” of chronic psychiatric diseases during development and its effects on major behavioral and brain abnormalities^82^. Our results also suggest that when the offspring was exposed to a “second-hit” stressor (postweaning CAF diet), behavior and brain volume might contrast from the fetal programming subjects, confirming a postnatal regulation of metabolic and behavioral traits by external stimuli. In this context, plasma lipidomic analysis of the offspring of mothers exposed to CAF diet showed a plasma decrease in the 22:6 lipid specie (n-3 PUFA), whereas an increase in the 20:4 specie (n-6 PUFA) (data unpublished). Some reports have identified lower levels of the n-3 polyunsaturated fatty acids (PUFA), eicosapentaenoic acid and docosahexaenoic acid whereas higher levels of the n-6 PUFA and araquidonic acid in the blood of subjects showing depressive or anxiety symptoms^83^. Notably, low levels of 22:6 lipid specie has been reported in the post mortem orbitofrontal cortex of patients with major depressive subjects^84^. Again, these evidence confirm the effect of fetal programming by high-fat diet on metabolic and behavioral traits in the offspring.

Our findings suggest that caloric diet programming reduces motivation for natural rewards, which relates with lower brain volume in the lateral hypothalamus and in the right NAc core showing defects in synaptophysin expression. This supports the role of CAF diet during gestation and lactation on setting a depression-like phenotype in young offspring.

## Supplementary information

Supplemental material
