# Supplementary material for: Maternal cafeteria diet exposure primes depression-like behavior in the offspring evoking lower brain volume related to changes in synaptic terminals and gliosis
Source: Transl Psychiatry. 2021 Jan 14;11:53. doi: 10.1038/s41398-020-01157-x (PMC7809040; doi:10.1038/s41398-020-01157-x)
Supplement: Supplementary file 1 — Supplemental material [file 41398_2020_1157_MOESM1_ESM.docx]

**Supplemental Information**

**Maternal cafeteria diet exposure primes depression-like behavior in the offspring evoking lower brain volume related to changes in synaptic terminals and gliosis**

CONTENTS

**Materials**

| Chocolate pellets (45mg), BioServ/ F05472). |
| --- |
| Chow diet (LabDiet/ No. 5001). |
| Cafeteria diet (CAF) with 372 Kcal/100g: 39% carbs, 49% lipids and 12% protein. |
| Paraformaldehyde (SIGMA-ALDRICH P6148-1KG) |
| Phosphate Buffered Saline (SIGMA P4417-100TAB) |
| Tritón X-100 (SIGMA T9284-100ML) |
| ProHance Gadoteridol 0.5M (BRACCO) |
| Anti-NR1 Rabbit antibody (Cell Signaling. 5704) |
| Anti-NMDAR2A Antibody (Invitrogen, 480031) |
| Anti-GluR2 Rabbit antibody (Cell Signaling. 5306) |
| Anti-GluR1 Rabbit antibody (Cell Signaling. 13185) |
| Anti-mGluR-2 (A-7). (Santa Cruz Biotech. sc-271655) |
| Anti-mGluR-5 (N-14): (Santa Cruz Biotech. sc-47147) |
| Anti-synaptophysin antibody (abcam, ab14692) |
| Anti-beta actin antibody (Cell Signaling, 8457) |
| Anti-rabbit IgG, HRP-linked antibody (Cell Signaling. 7074S) |
| Anti-mouse IgG, HRP-linked Antibody (Cell Signaling, 7076) |
| Mouse and Rabbit Specific HRP/DAB (ABC) Detection IHC Kit (abcam, ab64264) |
| Fomblin (SIGMA-ALDRICH 317926-100G) |

**Supplemental Methods**

**Rat acclimation and food restriction**

Upon arrival, females (n = 27) 10 weeks old and males (n = 97) 12 weeks old Wistar rats were group-housed for at least 10 days to acclimatize with *ad libitum* access to standard Chow diet and water. Animals were handled according to the NIH guide for the care and use of laboratory animals (NIH Publications No. 80–23, revised in 1996), with approval of the local Animal Care Committee (BI0002). All efforts were made to minimize the number of animals used and their suffering. Rats were housed individually in Plexiglas style cages, maintained at 22–23 °C and 12-h light/dark cycle.

# Maternal nutritional programming model in offspring

Females rats (n = 27) were randomized into two dietary groups: Control Chow and CAF diet, as we reported (1). Diet formulation was: Control Chow contained a caloric density of 3.35 kcal/g divided in 71% carbohydrates, 11% lipids and 18% proteins (Research Diets, New Brunswick, NJ, Cat. D12450B). Cafeteria (CAF) diet was made of liquid chocolate, biscuits, bacon, fried potatoes, standard diet and pork pate based on a 1:1:1:1:1:1:2 ratio, respectively; total calories 3.72 kcal/g in 39% carbohydrates, 49% lipids, 12% proteins and 513.53 mg of Sodium. After randomization, female rats were fed for 9 weeks, including 3 weeks of pre-pregnancy, pregnancy and lactation**,** respectively. Rats were mated with 12–14 weeks old Wistar males, 300–350 g, for two days. We registered female vaginal plug as a possible mating day (Figure S1).

Male offspring from mothers exposed to Chow or CAF diets were weaning by post-natal day 21, grouped into 10–12 subjects per group and were allocated into three groups according to the maternal programming diet. Offspring from mothers exposed to control Chow diet was kept under Chow diet (Group 1). Offspring from mothers exposed to CAF diet were kept under CAF diet (Group 2) or swift to control Chow diet (Group 3). Behavioral phenotyping was characterized at 8 weeks of age.

**Behavioral Phenotyping**

# *Preference to sucrose test*. The offspring was singly housed, and two drinking troughs were placed in the cages, one containing water and the second containing 2% w/v sucrose solution. Basal sucrose intake was measure for 72 hours under *ad libitum* food and water exposure. Sucrose preference was performed by quantifying water and 2% w/v sucrose intake for 20 min after food and water deprivation for 16 hours. The percentage of preference for sucrose in the offspring was quantified (see formula below) and baseline intake vs the test day was compared.

# % PS = [IS ÷ (SI + WI)] × 100.

PS: Preference to sucrose; SI: Sucrose intake; WI: Water intake

We decided to select the 2% w/v sucrose solution concentration to avoid differences in perception by low concentration. Based on our analysis, we propose that reduction of sucrose consumption in the offspring of fetal programmed groups is not an effect of sensorial traits.

# *Novelty suppressed feeding.* Rats were deprived of food for 18 hours; a Chow diet pellet was placed in the center of the open field arena (50 x 50 x 50 cm). The test was performed placing the subjects at the corner of the arena and we quantified the time to reach the Chow diet pellet. Subjects that were unable to reach the pellet in 5 minutes were not included in the analysis. Rats that reached the pellet were singly housed and were exposed to pre-weighted Chow food diet. We quantified food intake for the next 20 min.

***Open field test.*** In order to evaluate the anxiety behavior, offspring was handled by the base of their tails and placed in one of the four corners of the open field arena. Subjects were allowed to explore the apparatus for 5 minutes. After the 5-minute test, rats were returned to their home cages and the open field area was cleaned using 70 % ethyl alcohol. Activity was video recorded for 5 minutes using an automatic motion sensor system (OMNIALVA, Inc). We quantified the total distance traveled, the percentage of inactivity, time spent in the center and on the edges of the arena.

The behaviors scored (Brown et al, 1999) included: 1. Line Crossing: Frequency with which the mice crossed one of the grid lines with all four paws. 2. Center Square Entries: Frequency with which the mice crossed one of the red lines with all four paws into the central square. 3. Center Square Duration: Duration of time the mice spent in the central square.

***Operational Conditioning in the Skinner box****. Motivation for food intake was determined by operational conditioning using Skinner box, as reported previously (2, 3).*  After acclimation, rats were subsequently singly-housed, and food was restricted by lowering to 70% their daily chow food intake until they reach 90–95% body weight. This manipulation allowed acquisition of lever-press responding. Upon reaching a 5–10% weight loss daily food intake was adjusted to stabilize their lower body weights for the remainder of the training period.

*Fixed ratio (FR)-1 schedule.* A day prior to the start of the training session 10–15 high-fat and high-sugar (HFHS) dust-free precision food pellets (45 mg also available) containing 48.9% Kcal as fat (Bio Serv, Frenchtown, NJ) were placed in the home cage to prevent food neophobia on operant performance. Rats were trained to press the lever on a fixed ratio (FR)-1 reinforcement schedule where a single lever press delivers a food pellet to the receptacle. Only one lever is designated as “active” showing a 5 s timeout (TO) to the FR1 schedule (FR1/TO-5), during which additional lever-pressing does not result in the delivery of a food pellet. Each FR training session lasts 1 h or when 100 pellets have been delivered. We established that the animals got the acquisition criteria for food-maintained operant responses by validating if rats showed: (1) a minimum number of active responses and rewards earned and (2) 40 rewards per session over three consecutive days. As reported by Sharma et al., 2012, we have observed that∼75% of Wistar rats achieve acquisition criteria by 7–10 days of training. Rats that did not get acquisition criteria were excluded from the study. At this time, rats were returned to the *ad libitum* access to standard chow and water.

*Fixed ratio (FR)-5 schedule.* Following three successive training sessions of obtaining≥40 high fat high sugar (HFHS) pellets or until 100 pellets were obtained. We established the FR5/TO-5 s schedule where 5 active lever presses triggered the delivery of the food pellet. Training on the FR5 schedule lasted three days. As for FR1/TO-5, schedule rats were exposed to *ad libitum* access to standard chow and water.

*Progressive ratio (PR) schedule.* The PR testing was calculated as per Richardson and Roberts (1996) using the following formula (rounded to the nearest integer): = [5e(R*0.2)]−5 where R is equal to the number of food rewards already earned plus 1 (i.e., next reinforcer) (4). Thus, the number of responses required to earn a food reward follow the order: 1, 2, 4, 6, 9, 12, 15, 20,25, 32, 40, 50, 62, 77, 95, and so on. The final ratio completed is the breakpoint. We calculated the breakpoint of each experimental group as a maximal efficient response to food reward. As for FR schedules, the PR session lasts a maximum of 1 h per day. Failure to press the lever in any 10 min period results in termination of the session. We also verified performance on the PR schedule by documenting the stable reinforcement for food when the number of rewards earned in a 1 h session deviates by≤10% for at least 3 consecutive days. As for the FR1/TO-5and FR5/TO-5 schedule, rats were exposed to *ad libitum* access to standard chow and water. Breaking point was determined by the number of lever presses necessary for delivery of the last pellet obtained by the animals at the end of the session.

Following this procedure, we performed the behavioral phenotyping of two cohorts of offspring exposed to Chow or CAF diet during pregnancy and lactation. We used the first cohort to characterize macro and microplasticity using magnetic resonance imaging and immunohistochemistry and brain morphological analysis; and in the second cohort, we performed western blot analysis for synaptic markers.

# Intracardiac perfusion

# The offspring from cohort one was sacrificed by of intraperitoneally 1 mL pentobarbital (PiSA Agropecuaria) overdose. A dermal dissection was performed from the abdominal region to the upper part of the thoracic cage, exposing the heart. Then, left ventricle of the heart was perforated following its apex and a cut was made in the right atrium, to open the circulatory system. PBS 0.1M + Heparin + PROhance wash solution (4 mM) was perfused using an infusion pump (Fisher Scientific GP1000) at a flow rate of 10 ml / min. Subsequently, the washing solution was changed to the fixing solution including 4% paraformaldehyde in PBS 0.1M (PFA) + 4 mM Prohance for 25 min, following the same flow. The brain protected by the skull was collected and samples were stored at 4 ° C in 4% PFA Prohance 4 mM for 24 hours and then the solution was changed to 0.1M PBS with 0.02% sodium azide until further analysis in the MRI.

# Magnetic resonance imaging (MRI) analysis by morphometry based on deformation

# For MRI acquisition, the skulls were submerged and fixed inside plastic tubes filled with Fomblin (a chemically inert perfluoropolyether fluorocarbon; Solvay Solexis, Inc.). Imaging was performed in a 16 cm bore 7 T Bruker scanner (Pharmascan 70/16) using a Paravision 6.0.1 system and a Tx/Rx volume coil for rats with a 72 mm inner diameter. We then acquired a T1w sequence with the following parameters: Bruker FLASH, slice thickness = 0.0853 mm, TR/TE = 30.76/8.64 ms, flip angle = 20 degrees, averages = 1, matrix = 376 x 376, spacing = 0.0853 mm, Pixel bandwidth = 74 Hz, FOV = 300 x 300 mm, no of slices = 376.

# Morphological analysis

# All images were converted from DICOM to MINC format, and then preprocessed using an in-house pipeline based on MINC-Tools and ANTs, which performed the following steps: center image, full mask and N4 Bias Field Correction. We then used an image registration-based approach to assess anatomical differences between groups. Image registration finds a smooth spatial transformation that best aligns one image to another such that corresponding anatomical features are superimposed. We used an automated intensity-based group-wise registration approach (5) to align all brains in the study into a common coordinate system, yielding an average image of the 48 T1w scans. The deformation that alignments the images becomes a summary of how they differ. To assess volume differences between groups, we performed deformation-based morphometry as it provides a continuous voxel by voxel definition of volume changes (expansion/contraction) related to their mother’s and own exposure. Deformations were then mapped from the individual scans back to the average image. The final deformation fields were computed with a greedy symmetric diffeomorphic registration (the SyN algorithm in ANTS) (6,7) then inverted and blurred with a 0.1 mm FWHM Gaussian smoothing kernel. The Jacobian determinants of these deformations were extracted, giving a measure of local volume expansion/contraction at every voxel in the brain (8). Log-transformed Jacobian determinants (blurred 0.2 mm) were used to assess differences between groups because they better estimate a normal distribution (9). Details about the pipeline commands are available as open access (https://github.com/egarza/fetalratcaf_open).

# Histological analysis

# Brains from the MRI analysis were collected, dissected from the skull and washed with 0.1 M PBS, transferred to 50, 70, 80, 95 and 100% ethanol (10 min each), covered in paraffin and 5 μm coronal sections from the rostral-caudal direction were obtained in a cryostat. This strategy allowed us to characterized brain regions including the NAc, the Hippocampus and the PFC, according to the rat brain atlas Paxinos, George, and Charles Watson 2006. Brains sections were exposed to Xilol, ethanol and distilled water for hydration (2 min each). Sections were processed for Histological evaluation using Hematoxilyn-Eosin staining, Kluver-Barrier staining and immunohistochemistry (see below).

# *Histologic staining.* was performed to evaluate cells in the hippocampus and dentate gyrus of the 3 experimental groups. Importantly, all sections were processed similarly to ensure standard staining conditions. We examined the sections under a microscope (LAS EZ ver 3.0 by Leica). The images were captured by a digital camera (ICC50 HD). The average number of neurons contained in the dentate gyrus areas (354 x 265 µm) was calculated for each group. We distinguished neurons according to size and presence of Nissl bodies.

*Hematoxylin and eosin staining.* The sections were de-paraffined following the sequence of Xylene (15 minutes), absolute ethanol and Xylene 1: 1 (10 minutes), absolute ethanol (5 minutes), ethanol 96% (5 minutes) and finally distilled water. The slices were stained in hematoxylin solution for 8 minutes and then washed for 5 minutes in running tap water. Then, the slices were differentiated in 1% acid alcohol for 30 seconds and washed with running tap water for 1 minute. Next, they were submerged for 30 second in ammonia water and saturated lithium carbonate solution for 30 second. Finally, the slices were washed with running tap water and counterstain in eosin-phloxine solution for 5 minutes. At the end, the sections were dehydrated through 95% alcohol, 2 changes of absolute alcohol for 5 minutes each and cleared in 2 changes of xylene for 5 minute each. The slices were mounted with xylene based mounting medium.

*Kluver-Barrier staining.* The sections were de-paraffined following the sequence of Xylene (15 minutes), absolute ethanol and Xylene 1: 1 (10 minutes), absolute ethanol (5 minutes) and finally ethanol 96% (5 minutes). The slices were incubated overnight in 0.1% Luxol blue solution at room temperature. Subsequently, they were rinsed in 96 alcohol to remove excess dye and in distilled water until it stops fading. The differentiation in alcohol of 70 was carried out until the gray and white matter are distinguished. Finally, they were incubated with cresyl violet solution for 10 minutes and then washed in alcohol 96 and dehydrated in absolute alcohol and xylene.

# *Immunohistochemistry.* Antigen retrieval was performed by placing the sections into citrate buffer (10mM Citric Acid, 0.05% Tween 20, pH 6.0) at 90 ° C for 20 minutes. The slices were 2 x washed with 0.1 M PBS + 0.1% TritonX-100, incubated with the peroxidase blocker for 10 minutes (Abcam, Cat. AB64264), washed with 0.1M PBS + 0.1% TritonX-100 and incubated with the protein blocker for 10 minutes (Abcam, Cat. AB64264). Sections were incubated with 0.1M PBS + 0.1% TritonX-100, following by the primary anti-synaptophysin antibody (1: 200, Abcam. Cat. ab14692) at 4 ° C overnight. Finally, sections were exposed to biotinylated goat anti-rabbit IgG secondary antibody for 10 minutes, following by streptavidin (10 min) and diaminobenzidine DAB chromogen solution (30μL in 1.5mL of substrate) for 10 minutes. Hematoxylin staining was performed as contrast staining. Assembly of the cut was performed with mounting solution on coverslips. Negative controls were prepared by omission of primary antibodies and no densitometry signal was identified.

# Membrane and cytoplasmic fractions isolation from the brain samples

# Brains from the second cohort of subjects were dissected from the skull and the NAc, the PFC and the hippocampus were isolated using the Paxinos and Watson atlas as we reported previously (10). Brain regions were incubated in lysis buffer containing: 150 mM NaCl, 25 mM Tris-HCl pH 7.5, containing 50 mM NaF2, 10 mM NaP2O7, 1 mM sodium orthovanadate, cocktail of complete protease inhibitor and 0.5% Triton X-100 and homogenized by sonication (5 s to 1500 Hz on ice). Next, samples were centrifuged for 10 min x 1500 rpm and the protein concentration was determined by Bradford assay. Finally, supernatant (3 mg of protein in 500 μL per sample) was incubated for 30 min at 4 ° C and centrifuged for 20 min at 16,000 × g at 4 ° C, to separate a soluble extract in Triton and the insoluble pellet. The pellets were resuspended in 100 μl of lysis buffer and the protein concentration was determined by the Bradford assay, as we reported (10).

**Western blot analysis**

# Samples were mixed with Laemmli buffer, heated at 90 ° C for 5 min and subjected to sodium dodecyl sulfate polyacrylamide gel electrophoresis (SDS-PAGE) and then transferred to immobilon-P membrane (Millipore, Bedford, MA, USA) at 25 mA during 24 h at 4 °C. Membranes were blocked for 1 hour at room temperature with 5% Bovine Albumin in TBS-T buffer (10 mM Tris, 0.9% NaCl, 0.1% Tween 20, pH 7.5). The membranes were incubated overnight at 4 ° C with primary antibodies: anti-NMDA antibody, anti-AMPA, anti-synaptophysin, mGlur2 and mGlur5 (dilution 1: 1000, 1: 1000, 1: 500, 1:500 and 1:500 respectively). Finally, membranes were washed in TBS-T (4 times x 5 min) and incubated with secondary antibody conjugated anti-rabbit or goat peroxidase conjugated antibody (1: 1000) for 1 h. Membranes were washed in TBS-T (4 times x 10 min), incubated with chemiluminescence’s reagent (ECLTM Amersham Biosciences, Little Chalfont, UK) and detected the ChemiDoc (XRS System BioRad). Negative controls were prepared by omission of primary antibodies. Densitometry analysis was corrected with respect to actin content (1:10000, Cell signaling) and quantified by the 1.31V ImageJ software.


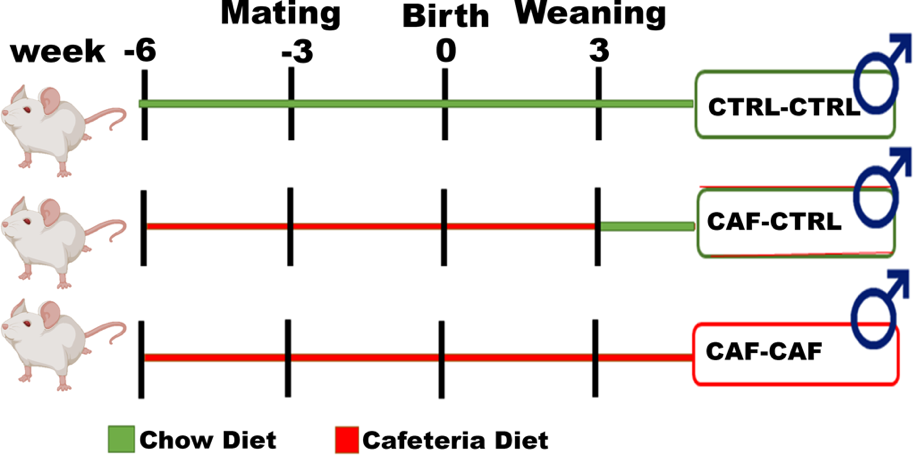
**Supplemental Figures**

*Figure S1. Fetal programming model. Females rats (n = 27) were randomized and were exposed to two dietary formula: Control Chow or CAF diet for 9 weeks, including 3 weeks of pre-pregnancy, pregnancy and lactation. Rats were mated with 12–14 weeks old Wistar males, 300–350 g, for two days. After weaning offspring was swift to Control Chow or kept on CAF diet exposure.*


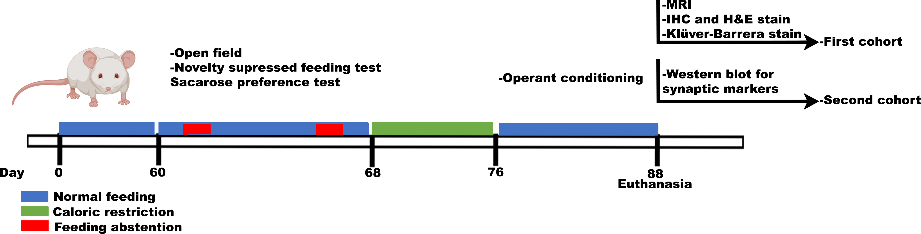


*Figure S2. Offspring after programming was behavioral tested using the open field, novelty suppressed feeding, sucrose preference and operant conditioning. Afterwards, we allocated the subjects into two cohorts. The first cohort to characterize macro and microplasticity using magnetic resonance imaging and immunohistochemistry and brain morphological analysis using H-E and Klúver-Barrera; and in the second cohort, we performed western blot analysis for synaptic markers.*

*
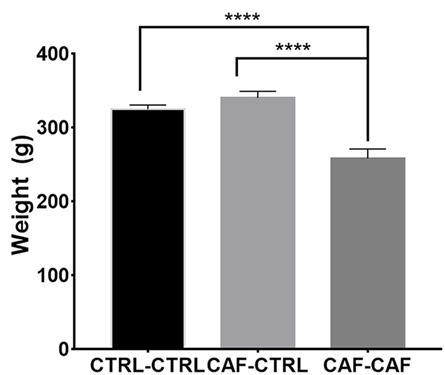
*

*Figure S3. The weight was measured at day 88 postnatal before the euthanasia. Nutritional programming by CAF diet decreased offspring weight (F2,49 = 20.88, p= 0.0005). only if the offspring continues the CAF diet after weaning (CTRL-CTRL vs CAF-CAF ****p= <0.0001). Results are expressed as mean + SEM, following by ANOVA post hoc Bonferroni Tukey *** p<0.05 vs the control*

*
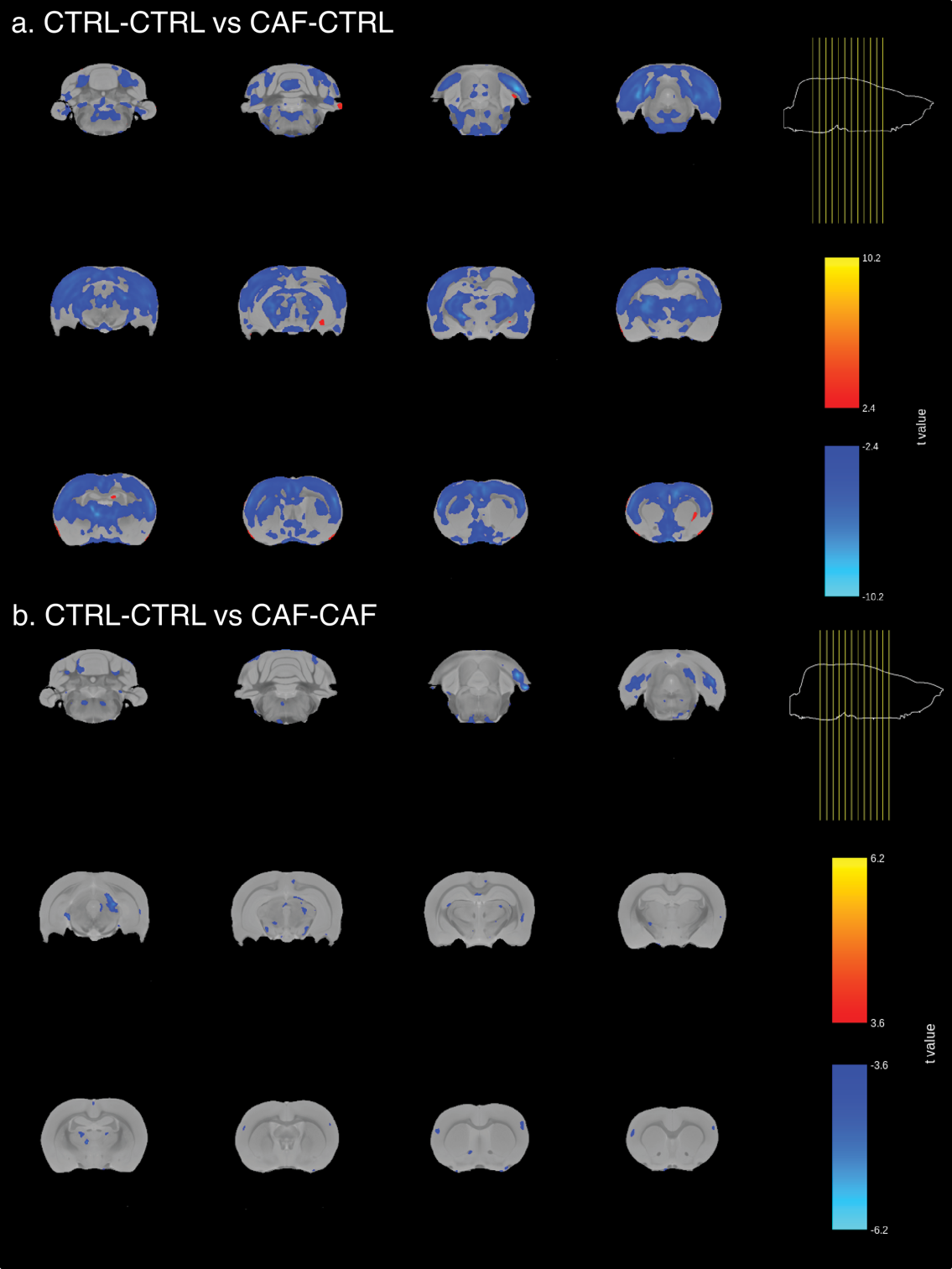
*

*Figure S4. Main Deformation-based morphometry (DBM) results. A) CN-CN vs CAF-CN between-subjects contrast results; B) CTRL-CTRL vs CAF-CAF between subject contrast results. Slices in coronal view. CTRL-CTRL (control-control); CAF-CTRL (maternal Cafeteria diet-control diet); CAF-CAF (maternal and offspring Cafeteria diet). Blue-light blue = lower volume; red-yellow = higher volume. Results are significant at FDR 5%.*


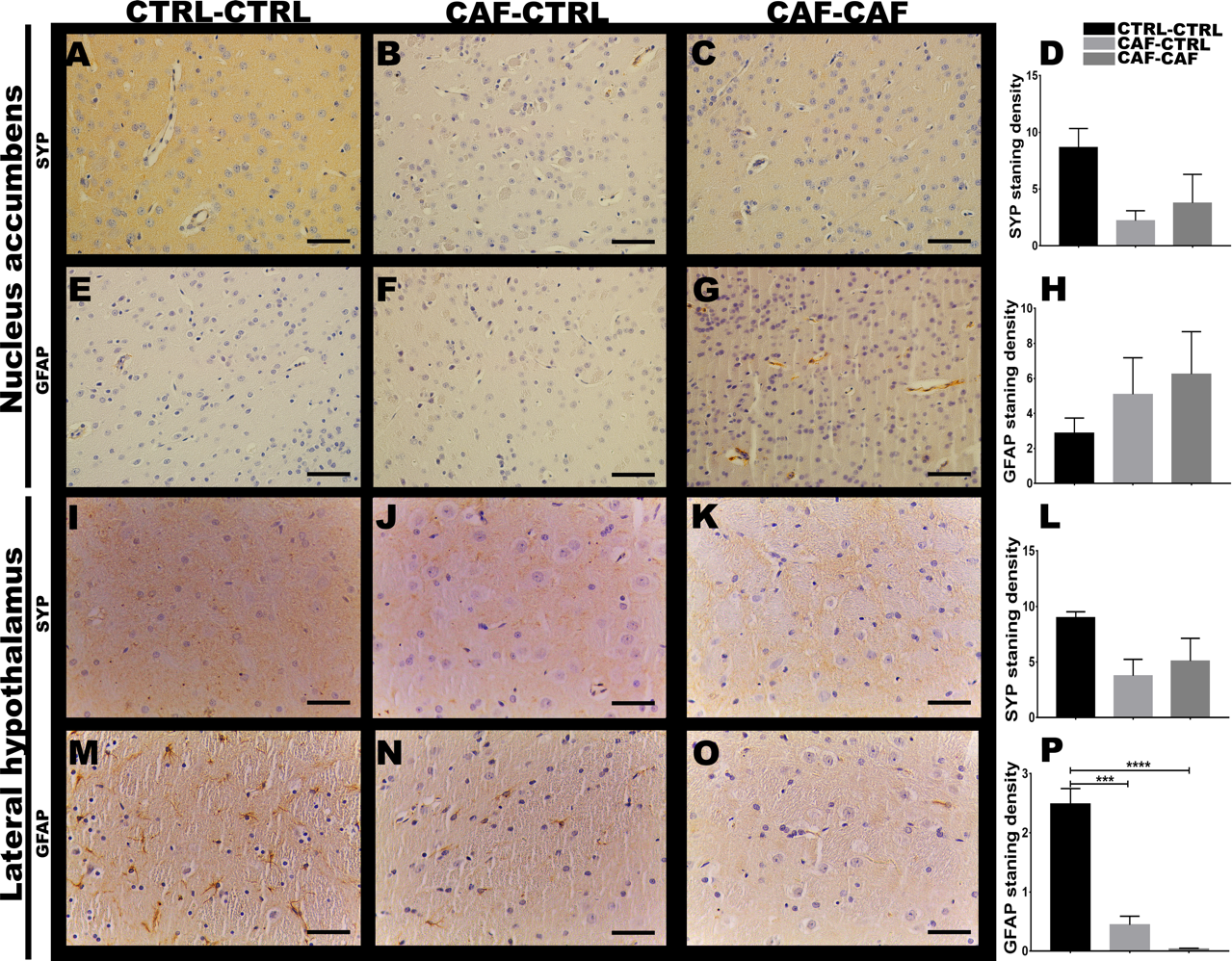


*Figure S5. Synaptophysin and GFAP expression in NAc and lateral hypothalamus in depression-like behavior subjects. Slices were obtained from brain previously scanned by MRI as described. Immunostaining of sections using anti-SYP (A-D) and anti-GFAP (E-H) antibodies in NAc. Immunostaining of sections using anti-SYP (I-L) and anti-GFAP (M-L) antibodies in lateral hypothalamus. Results are expressed as mean + SEM. following by ANOVA post hoc Tukey. ***P < 0.001, ****P < 0.0001 vs the control group (n= 4 per group). Scale bar = 50 μm.*

**Supplemental Tables**

**Table S1**

Control Chow and CAF diet formulas and caloric density

| Compound | Chow diet | Cafeteria diet |
| --- | --- | --- |
| Caloric density | 3.35 Kcal/g | 3.72 Kcal/g |
| Carbohydrates | 71% | 39% |
| Lipids | 11% | 49% |
| Protein | 18% | 12% |

^Cafeteria (CAF) diet was made of liquid chocolate, biscuits, bacon, fries’ potatoes, standard diet and pork pate based on a 1:1:1:1:1:1:2 ratio, respectively^

**Table S2**

Responders population portion between experimental offspring groups after operant conditioning test

| Experimental group | High responders (%) | Low responders (%) |
| --- | --- | --- |
| CTRL-CTRL | 81* | 19 |
| CAF-CTRL | 53 | 47 |
| CAF-CAF | 65 | 35 |

^Classified based on the number of events obtained in PR; Chi2 test *p< 0.05 for high vs low responders in the CTRL-CTRL. n=19/group^

**Table S3**

Areas affected by tissue enlargement and shrinkage after a maternal programming (CAF-CTRL group)

| **Brain region** | **Side** | **t-stat** | **Volume change** | **Coordinates** | | |
| --- | --- | --- | --- | --- | --- | --- |
| Frontal Association Cortex | R | 5.57 | Higher | 3 | 6 | 0.5 |
| Frontal Association Cortex | L | 5.125 | Higher | -5 | 6 | 2.5 |
| Piriform Cortex | L | 5.124 | Higher | -6 | 0.3 | -0.4 |
| Cerebellar lobule 6 | L | 4.368 | Higher | -0.04 | -10 | 7 |
| Primary Somatosensory Cortex | R | 3.273 | Higher | 6.3 | 1.5 | 3.8 |
| Hippocampal layer CA3 | R | 2.957 | Higher | 1.3 | 0.4 | 4.2 |
| Rhinal cortex | R | -10.3 | Lower | 3.8 | -5.5 | 3.7 |
| Occipital Cortex | L | -9.357 | Lower | -4.8 | -5.5 | 4 |
| Frontal Association Cortex | L | -8.826 | Lower | -.2 | 6.7 | -0.6 |
| Hypothalamus | L | -8.4 | Lower | -2.7 | -0.8 | 0.9 |
| Olfactory Bulb | L | -7.715 | Lower | -1.2 | 11.9 | 1.6 |
| Primary Somatosensory Cortex | R | -7.512 | Lower | 5.4 | 1.6 | 4 |
| Secondary Motor Cortex | R | -7.276 | Lower | 0.7 | 4.9 | 4.9 |
| Retrosplenial Cortex/cingulum | R | -6.264 | Lower | 0.04 | -0.4 | 6.3 |

^Repeated measure ANOVA at a threshold of p<0.001 (FDR corrected); t-test for volume increase and decrease in the CAF-CTRL group compared to the CTRL-CTRL group.^

**Table S4**

Areas affected by tissue shrinkage after a maternal programming and following maternal diet (CAF-CAF group)

| **Brain region** | **Side** | **t-stat** | **Volume change** | **Coordinates** | | |
| --- | --- | --- | --- | --- | --- | --- |
| Arbor vita of cerebellum | L | -6.223 | Lower | -0.3 | -9.6 | 5.5 |
| Thalamus | L | -6.107 | Lower | -1.2 | 0.1 | 1.3 |
| Occipital Cortex | R | -5.886 | Lower | 4.1 | -5.6 | 3.6 |
| Hindbrain/medulla/Pons | L | -5.226 | Lower | -1.3 | -6.7 | -1.8 |
| Crus 1 ansiform lobule | L | -5.019 | Lower | -5.4 | -7.7 | 6 |
| Rhinal cortex | L | -4.741 | Lower | -4.1 | -4.2 | 2.6 |
| Olfactory tubercle | R | -4.56 | Lower | 2.5 | 2.9 | -2 |
| Paramedian lobe | L | -4.456 | Lower | -4.6 | -10 | 1.7 |
| Primary Somatosensory Cortex | L | -4.408 | Lower | -5.7 | 3.3 | 3.1 |
| Hippocampal layer CA1 | R | -4.382 | Lower | 2.8 | -2.1 | -0.8 |
| Retrosplenial Cortex | L | -4.353 | Lower | -0.4 | 0.8 | 6.7 |
| Primary Somatosensory Cortex | R | -3.964 | Lower | 5.3 | 2.6 | 4.2 |

^Repeated measure ANOVA at a threshold of p<0.001 (FDR corrected); t-test for volume increase and decrease in the CAF-CAF group compared to the CTRL-CTRL group.^

For full MRI data of the current study are freely available at: https://zenodo.org/record/4279989#.X7XQw3b0k5k

**Raw data of Western Blots**
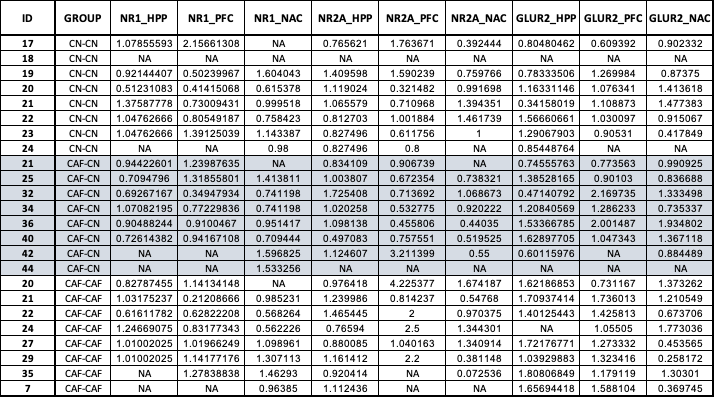


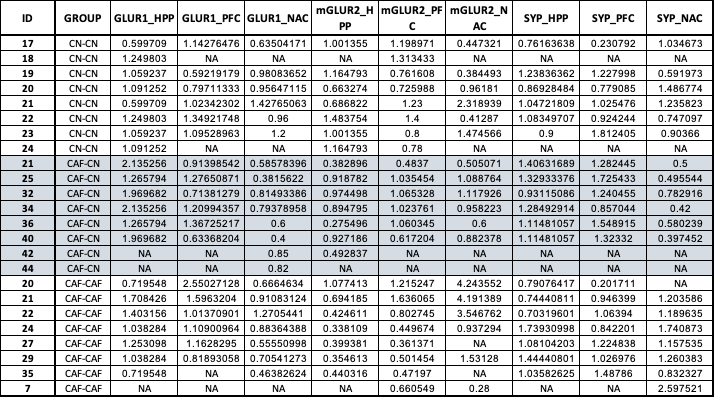


**Statistics for Western Blot analysis**

| Fig. | Parameter | N | Data structure | Comparison | Cl_95_ | Type of test | p value |
| --- | --- | --- | --- | --- | --- | --- | --- |
| 1-A | [A] HR CTRL-CTRL | 13 | Normal Distribution | A vs B | 20.95 to 57.66 | Two-way ANOVA with Holm-Sidak´s multiple comparison | <0.0001 |
|  | [B] LR CTRL-CTRL | 3 | Normal Distribution | A vs C | -3.120 to 21.73 | Two-way ANOVA with Holm-Sidak´s multiple comparison | 0.0190 |
|  | [C] HR CAF-CTRL | 9 | Normal Distribution | A vs D | 26.85 to 50.96 | Two-way ANOVA with Holm-Sidak´s multiple comparison | <0.0001 |
|  | [D] LR CAF-CTRL | 10 | Normal Distribution | A vs E | -0.4330 to 23.05 | Two-way ANOVA with Holm-Sidak´s multiple comparison | 0.0170 |
|  | [E] HR CAF-CAF | 11 | Normal Distribution | A vs F | 24.00 to 52.29 | Two-way ANOVA with Holm-Sidak´s multiple comparison | <0.0001 |
|  | [F] LR CAF-CAF | 6 | Normal Distribution | B vs C | -49.11 to -10.89 | Two-way ANOVA with Holm-Sidak´s multiple comparison | 0.0002 |
|  |  |  |  | B vs D | -19.27 to 18.47 | Two-way ANOVA with Holm-Sidak´s multiple comparison | 0.9975 |
|  |  |  |  | B vs E | -46.67 to -9.333 | Two-way ANOVA with Holm-Sidak´s multiple comparison | 0.0004 |
|  |  |  |  | B vs F | -21.43 to 19.10 | Two-way ANOVA with Holm-Sidak´s multiple comparison | 0.9975 |
|  |  |  |  | C vs D | 16.43 to 42.77 | Two-way ANOVA with Holm-Sidak´s multiple comparison | <0.0001 |
|  |  |  |  | C vs E | -10.88 to 14.88 | Two-way ANOVA with Holm-Sidak´s multiple comparison | 0.9844 |
|  |  |  |  | C vs F | 13.73 to 43.94 | Two-way ANOVA with Holm-Sidak´s multiple comparison | <0.0001 |
|  |  |  |  | D vs E | -40.12 to -15.08 | Two-way ANOVA with Holm-Sidak´s multiple comparison | <0.0001 |
|  |  |  |  | D vs F | -15.57 to 14.03 | Two-way ANOVA with Holm-Sidak´s multiple comparison | 0.9975 |
|  |  |  |  | E vs F | 12.29 to 41.38 | Two-way ANOVA with Holm-Sidak´s multiple comparison | <0.0001 |
| 1-B | [A] CTRL-CTRL | 13 | Normal Distribution | A vs B | -7.763 to 1.840 | One-way ANOVA with Tukey´s multiple comparison | 0.2958 |
|  | [B] CAF-CTRL | 12 | Normal Distribution | A vs C | -11.23 to -0.4469 | One-way ANOVA with Tukey´s multiple comparison | 0.0316 |
|  | [C] CAF-CAF | 9 | Normal Distribution | B vs C | -8.350 to 2.600 | One-way ANOVA with Tukey´s multiple comparison | 0.4091 |
| 1-C | [A] CTRL-CTRL BL | 6 | Normal Distribution | A vs B | -10.94 to 9.278 | Two-way ANOVA with Tukey´s multiple comparison | 0.9999 |
|  | [B] CAF-CTRL BL | 12 | Normal Distribution | A vs C | -10.79 to 9.733 | Two-way ANOVA with Tukey´s multiple comparison | >0.9999 |
|  | [C] CAF-CAF BL | 11 | Normal Distribution | A vs D | -2.012 to 22.48 | Two-way ANOVA with Tukey´s multiple comparison | 0.1511 |
|  | [D] CTRL-CTRL TEST | 5 | Normal Distribution | A vs E | 10.22 to 30.44 | Two-way ANOVA with Tukey´s multiple comparison | <0.0001 |
|  | [E] CAF-CTRL TEST | 12 | Normal Distribution | A vs F | 9.661 to 30.19 | Two-way ANOVA with Tukey´s multiple comparison | <0.0001 |
|  | [F] CAF-CAF TEST | 11 | Normal Distribution | B vs C | -8.138 to 8.744 | Two-way ANOVA with Tukey´s multiple comparison | >0.9999 |
|  |  |  |  | B vs D | 0.3025 to 21.83 | Two-way ANOVA with Tukey´s multiple comparison | 0.0406 |
|  |  |  |  | B vs E | 12.91 to 29.42 | Two-way ANOVA with Tukey´s multiple comparison | <0.0001 |
|  |  |  |  | B vs F | 12.32 to 29.20 | Two-way ANOVA with Tukey´s multiple comparison | <0.0001 |
|  |  |  |  | C vs D | -0.1435 to 21.67 | Two-way ANOVA with Tukey´s multiple comparison | 0.0550 |
|  |  |  |  | C vs E | 12.42 to 29.30 | Two-way ANOVA with Tukey´s multiple comparison | <0.0001 |
|  |  |  |  | C vs F | 11.83 to 29.08 | Two-way ANOVA with Tukey´s multiple comparison | <0.0001 |
|  |  |  |  | D vs E | -0.6642 to 20.86 | Two-way ANOVA with Tukey´s multiple comparison | 0.0015 |
|  |  |  |  | D vs F | -1.216 to 20.60 | Two-way ANOVA with Tukey´s multiple comparison | 0.0074 |
|  |  |  |  | E vs F | -8.850 to 8.032 | Two-way ANOVA with Tukey´s multiple comparison | >0.9999 |
| 1-D | [A] CTRL-CTRL | 6 | Normal Distribution | A vs B | -224.2 to -14.58 | One-way ANOVA with Tukey´s multiple comparison | 0.0234 |
|  | [B] CAF-CTRL | 10 | Normal Distribution | A vs C | -237.8 to -31.80 | One-way ANOVA with Tukey´s multiple comparison | 0.0088 |
|  | [C] CAF-CAF | 11 | Normal Distribution | B vs C | -104.1 to 73.27 | One-way ANOVA with Tukey´s multiple comparison | 0.9017 |
| 1-E | [A] CTRL-CTRL | 6 | Normal Distribution | A vs B | 0.1572 to 1.620 | One-way ANOVA with Tukey´s multiple comparison | 0.0150 |
|  | [B] CAF-CTRL | 10 | Normal Distribution | A vs C | 0.7835 to 2.562 | One-way ANOVA with Tukey´s multiple comparison | 0.0002 |
|  | [C] CAF-CAF | 11 | Normal Distribution | B vs C | -0.09175 to 1.660 | One-way ANOVA with Tukey´s multiple comparison | 0.0857 |
| 1-F | [A] CTRL-CTRL | 6 | Normal Distribution | A vs B | -8.185 to -0.3933 | One-way ANOVA with Tukey´s multiple comparison | 0.0288 |
|  | [B] CAF-CTRL | 8 | Normal Distribution | A vs C | -7.272 to -0.3656 | One-way ANOVA with Tukey´s multiple comparison | 0.0280 |
|  | [C] CAF-CAF | 16 | Normal Distribution | B vs C | -2.653 to 3.594 | One-way ANOVA with Tukey´s multiple comparison | 0.9263 |
| 1-G | [A] CTRL-CTRL | 6 | Normal Distribution | A vs B | 0.4112 to 29.77 | One-way ANOVA with Tukey´s multiple comparison | 0.0431 |
|  | [B] CAF-CTRL | 8 | Normal Distribution | A vs C | 1.833 to 28.36 | One-way ANOVA with Tukey´s multiple comparison | 0.0235 |
|  | [C] CAF-CAF | 13 | Normal Distribution | B vs C | -12.04 to 12.05 | One-way ANOVA with Tukey´s multiple comparison | >0.9999 |
| 1-H | [A] CTRL-CTRL | 6 | Normal Distribution | A vs B | -21.76 to -3.348 | One-way ANOVA with Tukey´s multiple comparison | 0.0063 |
|  | [B] CAF-CTRL | 8 | Normal Distribution | A vs C | -15.12 to 1.701 | One-way ANOVA with Tukey´s multiple comparison | 0.1358 |
|  | [C] CAF-CAF | 13 | Normal Distribution | B vs C | -1.817 to 13.50 | One-way ANOVA with Tukey´s multiple comparison | 0.1591 |
| 1-I | [A] CTRL-CTRL | 5 | Normal Distribution | A vs B | 3.467 to 23.31 | One-way ANOVA with Tukey´s multiple comparison | 0.0069 |
|  | [B] CAF-CTRL | 8 | Normal Distribution | A vs C | -2.131 to 16.00 | One-way ANOVA with Tukey´s multiple comparison | 0.1576 |
|  | [C] CAF-CAF | 14 | Normal Distribution | B vs C | -14.16 to 1.259 | One-way ANOVA with Tukey´s multiple comparison | 0.1132 |
| 5-B | [A] CTRL-CTRL | 6 | Normal Distribution | A vs B | -0.1781 to 0.4899 | One-way ANOVA with Tukey´s multiple comparison | 0.4644 |
|  | [B] CAF-CTRL | 6 | Normal Distribution | A vs C | -0.2938 to 0.3742 | One-way ANOVA with Tukey´s multiple comparison | 0.9478 |
|  | [C] CAF-CAF | 6 | Normal Distribution | B vs C | -0.4497 to 0.2183 | One-way ANOVA with Tukey´s multiple comparison | 0.6486 |
| 5-C | [A] CTRL-CTRL | 7 | Normal Distribution | A vs B | -0.4502 to 0.3143 | One-way ANOVA with Tukey´s multiple comparison | 0.8942 |
|  | [B] CAF-CTRL | 7 | Normal Distribution | A vs C | -0.4600 to 0.2802 | One-way ANOVA with Tukey´s multiple comparison | 0.8126 |
|  | [C] CAF-CAF | 8 | Normal Distribution | B vs C | -0.3920 to 0.3482 | One-way ANOVA with Tukey´s multiple comparison | 0.9876 |
| 5-D | [A] CTRL-CTRL | 7 | Normal Distribution | A vs B | -0.6397 to 0.4198 | One-way ANOVA with Tukey´s multiple comparison | 0.8579 |
|  | [B] CAF-CTRL | 7 | Normal Distribution | A vs C | -1.123 to -0.06366 | One-way ANOVA with Tukey´s multiple comparison | 0.0268 |
|  | [C] CAF-CAF | 8 | Normal Distribution | B vs C | -1.013 to 0.04629 | One-way ANOVA with Tukey´s multiple comparison | 0.0771 |
| 5-E | [A] CTRL-CTRL | 8 | Non Normal Distribution | A vs B |  | One-way ANOVA with Kruskal-Wallis´ multiple comparison | 0.0060 |
|  | [B] CAF-CTRL | 6 | Non Normal Distribution | A vs C |  | One-way ANOVA with Kruskal-Wallis´ multiple comparison | >0.9999 |
|  | [C] CAF-CAF | 7 | Non Normal Distribution | B vs C |  | One-way ANOVA with Kruskal-Wallis´ multiple comparison | 0.0347 |
| 5-F | [A] CTRL-CTRL | 7 | Normal Distribution | A vs B | -0.06033 to 0.7174 | One-way ANOVA with Tukey´s multiple comparison | 0.1065 |
|  | [B] CAF-CTRL | 7 | Normal Distribution | A vs C | 0.1022 to 0.8799 | One-way ANOVA with Tukey´s multiple comparison | 0.0125 |
|  | [C] CAF-CAF | 8 | Normal Distribution | B vs C | -0.2263 to 0.5514 | One-way ANOVA with Tukey´s multiple comparison | 0.5460 |
| 5-G | [A] CTRL-CTRL | 7 | Normal Distribution | A vs B | -0.3836 to 0.4770 | One-way ANOVA with Tukey´s multiple comparison | 0.9590 |
|  | [B] CAF-CTRL | 7 | Normal Distribution | A vs C | -0.1789 to 0.6544 | One-way ANOVA with Tukey´s multiple comparison | 0.3366 |
|  | [C] CAF-CAF | 8 | Normal Distribution | B vs C | -0.2256 to 0.6077 | One-way ANOVA with Tukey´s multiple comparison | 0.4876 |
| 5-H | [A] CTRL-CTRL | 6 | Normal Distribution | A vs B | -0.6231 to 0.1960 | One-way ANOVA with Tukey´s multiple comparison | 0.3917 |
|  | [B] CAF-CTRL | 6 | Normal Distribution | A vs C | -0.4883 to 0.3010 | One-way ANOVA with Tukey´s multiple comparison | 0.8155 |
|  | [C] CAF-CAF | 7 | Normal Distribution | B vs C | -0.2747 to 0.5145 | One-way ANOVA with Tukey´s multiple comparison | 0.7179 |
| 5-J | [A] CTRL-CTRL | 6 | Normal Distribution | A vs B | -0.6313 to 0.7873 | One-way ANOVA with Tukey´s multiple comparison | 0.9567 |
|  | [B] CAF-CTRL | 6 | Normal Distribution | A vs C | -0.5768 to 0.7902 | One-way ANOVA with Tukey´s multiple comparison | 0.9149 |
|  | [C] CAF-CAF | 7 | Normal Distribution | B vs C | -0.6548 to 0.7122 | One-way ANOVA with Tukey´s multiple comparison | 0.9936 |
| 5-K | [A] CTRL-CTRL | 7 | Normal Distribution | A vs B | -0.8106 to 1.407 | One-way ANOVA with Tukey´s multiple comparison | 0.7724 |
|  | [B] CAF-CTRL | 6 | Normal Distribution | A vs C | -2.378 to -0.2477 | One-way ANOVA with Tukey´s multiple comparison | 0.0149 |
|  | [C] CAF-CAF | 7 | Normal Distribution | B vs C | -2.720 to -0.5024 | One-way ANOVA with Tukey´s multiple comparison | 0.0045 |
| 5-L | [A] CTRL-CTRL | 6 | Normal Distribution | A vs B | -0.9497 to 0.2232 | One-way ANOVA with Tukey´s multiple comparison | 0.2772 |
|  | [B] CAF-CTRL | 6 | Normal Distribution | A vs C | -0.8376 to 0.2596 | One-way ANOVA with Tukey´s multiple comparison | 0.3874 |
|  | [C] CAF-CAF | 8 | Normal Distribution | B vs C | -0.4744 to 0.6228 | One-way ANOVA with Tukey´s multiple comparison | 0.9360 |
| 5-M | [A] CTRL-CTRL | 6 | Normal Distribution | A vs B | -0.6696 to 0.6313 | One-way ANOVA with Tukey´s multiple comparison | 0.9968 |
|  | [B] CAF-CTRL | 6 | Normal Distribution | A vs C | -1.026 to 0.2753 | One-way ANOVA with Tukey´s multiple comparison | 0.3196 |
|  | [C] CAF-CAF | 7 | Normal Distribution | B vs C | -1.006 to 0.2945 | One-way ANOVA with Tukey´s multiple comparison | 0.3553 |
| 5-N | [A] CTRL-CTRL | 8 | Normal Distribution | A vs B | -0.3315 to 0.6221 | One-way ANOVA with Tukey´s multiple comparison | 0.7230 |
|  | [B] CAF-CTRL | 6 | Normal Distribution | A vs C | -0.1776 to 0.7053 | One-way ANOVA with Tukey´s multiple comparison | 0.3048 |
|  | [C] CAF-CAF | 8 | Normal Distribution | B vs C | -0.3582 to 0.5954 | One-way ANOVA with Tukey´s multiple comparison | 0.8046 |
| 5-O | [A] CTRL-CTRL | 6 | Normal Distribution | A vs B | -0.6012 to 0.2964 | One-way ANOVA with Tukey´s multiple comparison | 0.6653 |
|  | [B] CAF-CTRL | 6 | Normal Distribution | A vs C | -0.4257 to 0.4139 | One-way ANOVA with Tukey´s multiple comparison | 0.9993 |
|  | [C] CAF-CAF | 8 | Normal Distribution | B vs C | -0.2734 to 0.5662 | One-way ANOVA with Tukey´s multiple comparison | 0.6508 |
| 5-P | [A] CTRL-CTRL | 6 | Normal Distribution | A vs B | -0.9472 to 0.2880 | One-way ANOVA with Tukey´s multiple comparison | 0.3756 |
|  | [B] CAF-CTRL | 6 | Normal Distribution | A vs C | -0.5657 to 0.6245 | One-way ANOVA with Tukey´s multiple comparison | 0.9911 |
|  | [C] CAF-CAF | 7 | Normal Distribution | B vs C | -0.2361 to 0.9541 | One-way ANOVA with Tukey´s multiple comparison | 0.2922 |
| 5-R | [A] CTRL-CTRL | 6 | Normal Distribution | A vs B | -0.6017 to 0.4390 | One-way ANOVA with Tukey´s multiple comparison | 0.9163 |
|  | [B] CAF-CTRL | 7 | Normal Distribution | A vs C | -0.5351 to 0.4751 | One-way ANOVA with Tukey´s multiple comparison | 0.9874 |
|  | [C] CAF-CAF | 8 | Normal Distribution | B vs C | -0.4327 to 0.5354 | One-way ANOVA with Tukey´s multiple comparison | 0.9605 |
| 5-S | [A] CTRL-CTRL | 6 | Normal Distribution | A vs B | -0.4085 to 0.9961 | One-way ANOVA with Tukey´s multiple comparison | 0.5428 |
|  | [B] CAF-CTRL | 7 | Normal Distribution | A vs C | -0.4541 to 0.8597 | One-way ANOVA with Tukey´s multiple comparison | 0.7128 |
|  | [C] CAF-CAF | 8 | Normal Distribution | B vs C | -0.7479 to 0.5659 | One-way ANOVA with Tukey´s multiple comparison | 0.9330 |
| 5-T | [A] CTRL-CTRL | 6 | Normal Distribution | A vs B | -0.8254 to 0.5160 | One-way ANOVA with Tukey´s multiple comparison | 0.8279 |
|  | [B] CAF-CTRL | 7 | Normal Distribution | A vs C | -0.5779 to 0.7242 | One-way ANOVA with Tukey´s multiple comparison | 0.9558 |
|  | [C] CAF-CAF | 8 | Normal Distribution | B vs C | -0.3961 to 0.8517 | One-way ANOVA with Tukey´s multiple comparison | 0.6277 |
| 5-U | [A] CTRL-CTRL | 6 | Normal Distribution | A vs B | 0.03790 to 0.7039 | One-way ANOVA with Tukey´s multiple comparison | 0.0278 |
|  | [B] CAF-CTRL | 8 | Normal Distribution | A vs C | -0.09584 to 0.5903 | One-way ANOVA with Tukey´s multiple comparison | 0.1854 |
|  | [C] CAF-CAF | 7 | Normal Distribution | B vs C | -0.4428 to 0.1954 | One-way ANOVA with Tukey´s multiple comparison | 0.5928 |
| 5-V | [A] CTRL-CTRL | 6 | Normal Distribution | A vs B | -1.529 to 1.812 | One-way ANOVA with Tukey´s multiple comparison | 0.9738 |
|  | [B] CAF-CTRL | 6 | Normal Distribution | A vs C | -3.125 to 0.2154 | One-way ANOVA with Tukey´s multiple comparison | 0.0427 |
|  | [C] CAF-CAF | 7 | Normal Distribution | B vs C | -3.267 to 0.07412 | One-way ANOVA with Tukey´s multiple comparison | 0.0421 |
| 5-W | [A] CTRL-CTRL | 6 | Normal Distribution | A vs B | -0.1369 to 1.078 | One-way ANOVA with Tukey´s multiple comparison | 0.1446 |
|  | [B] CAF-CTRL | 6 | Normal Distribution | A vs C | -1.011 to 0.1594 | One-way ANOVA with Tukey´s multiple comparison | 0.1773 |
|  | [C] CAF-CAF | 7 | Normal Distribution | B vs C | -1.482 to -0.3112 | One-way ANOVA with Tukey´s multiple comparison | 0.0031 |

**Supplemental References**

1. Cardenas-Perez RE, Fuentes-Mera L, De La Garza AL, Torre-Villalvazo I, Reyes-Castro LA, Rodriguez-Rocha H, et al. Maternal overnutrition by hypercaloric diets programs hypothalamic mitochondrial fusion and metabolic dysfunction in rat male offspring. Nutr Metab. 2018;15(1):1–16.

2. Camacho A, Montalvo-Martinez L, Cardenas-Perez RE, Fuentes-Mera L, Garza-Ocañas L. Obesogenic diet intake during pregnancy programs aberrant synaptic plasticity and addiction-like behavior to a palatable food in offspring. Behav Brain Res [Internet]. 2017;330(March):46–55. Available from: <http://dx.doi.org/10.1016/j.bbr.2017.05.014>

3. Cruz-Carrillo G, Montalvo-Martínez L, Cárdenas-Tueme M, Bernal-Vega S, Maldonado-Ruiz R, Reséndez-Pérez D, et al. Fetal Programming by Methyl Donors Modulates Central Inflammation and Prevents Food Addiction-Like Behavior in Rats. Front Neurosci. 2020;14(June):1–15.

4. Richardson, N. R., and Roberts, D. C. S. (1996). Progressive ratio schedules in drug self-administration studies in rats: a method to evaluate reinforcing efficacy. J. Neurosci. Methods 66, 1–11. doi: 10.1016/0165-0270(95)00153-0

5. Lerch JP, Sled JG, Henkelman RM. Magnetic resonance neuroimaging. In: Magnetic Resonance Neuroimaging, Methods in Molecular Biology. 2011. p. 349–61.

6. Avants B, Epstein C., Grossman M, Gee JC. Symmetric Diffeomorphic Image Registration with Cross- Correlation: Evaluating Automated Labeling of Elderly and Neurodegenerative Brain. Med Image Anal. 2008;48(Suppl 2):1–6.

7. Bird S, Klein E, Loper E. Natural Language Processing with Python. O´Really; 2009.

8. Chung MK, Worsley KJ, Paus T, Cherif C, Collins DL, Giedd JN, et al. A unified statistical approach to deformation-based morphometry. Neuroimage. 2001;14(3):595–606.

9. Leow AD, Klunder AD, Jr. CRJ, Toga AW, Dale AM, Bernstein MA, et al. Longitudinal stability of MRI for mapping brain change using tensor-based morphometry. Neuroimage. 2007;31(2):627–40.

10. Delint-Ramirez I, Maldonado Ruiz R, Torre-Villalvazo I, Fuentes-Mera L, Garza Ocañas L, Tovar A, et al. Genetic obesity alters recruitment of TANK-binding kinase 1 and AKT into hypothalamic lipid rafts domains. Neurochem Int [Internet]. 2015;80:23–32. Available from: <http://dx.doi.org/10.1016/j.neuint.2014.11.002>
